# Genome-wide identification of the *Liriodendron chinense WRKY* gene family and its diverse roles in response to multiple abiotic stress

**DOI:** 10.1186/s12870-021-03371-1

**Published:** 2022-01-10

**Authors:** Weihuang Wu, Sheng Zhu, Lin Xu, Liming Zhu, Dandan Wang, Yang Liu, Siqin Liu, Zhaodong Hao, Ye Lu, Liming Yang, Jisen Shi, Jinhui Chen

**Affiliations:** 1 Key Laboratory of Forest Genetics and Biotechnology, Ministry of Education of China, Co-Innovation Center for the Sustainable Forestry in Southern China, Nanjing Forestry University, Nanjing, China; 2 College of Biology and the Environment, Nanjing Forestry University, Nanjing, China

**Keywords:** *Liriodendron chinense*, Genome-wide, *WRKY* gene family, Transcription factor, Abiotic stress

## Abstract

**Background:**

*Liriodendron chinense* (*Lchi*) is a tree species within the *Magnoliaceae* family and is considered a basal angiosperm. The too low or high temperature or soil drought will restrict its growth as the adverse environmental conditions, thus improving *L. chinense* abiotic tolerance was the key issues to study. WRKYs are a major family of plant transcription factors known to often be involved in biotic and abiotic stress responses. So far, it is still largely unknown if and how the *LchiWRKY* gene family is tied to regulating *L. chinense* stress responses. Therefore, studying the involvement of the *WRKY* gene family in abiotic stress regulation in *L. chinense* could be very informative in showing how this tree deals with such stressful conditions.

**Results:**

In this research, we performed a genome-wide analysis of the *Liriodendron chinense* (*Lchi*) *WRKY* gene family, studying their classification relationships, gene structure, chromosomal locations, gene duplication, cis-element, and response to abiotic stress. The 44 members of the *LchiWRKY* gene family contain a significant amount of sequence diversity, with their lengths ranging from 525 bp to 40,981 bp. Using classification analysis, we divided the 44 *LchiWRKY* genes into three phylogenetic groups (I, II, II), with group II then being further divided into five subgroups (IIa, IIb, IIc, IId, IIe). Comparative phylogenetic analysis including the *WRKY* families from 17 plant species suggested that *LchiWRKYs* are closely related to the Magnolia *Cinnamomum kanehirae WRKY* family, and has fewer family members than higher plants. We found the *LchiWRKYs* to be evenly distributed across 15 chromosomes, with their duplication events suggesting that tandem duplication may have played a major role in *LchiWRKY* gene expansion model. A Ka/Ks analysis indicated that they mainly underwent purifying selection and distributed in the group IId. Motif analysis showed that LchiWRKYs contained 20 motifs, and different phylogenetic groups contained conserved motif. Gene ontology (GO) analysis showed that *LchiWRKYs* were mainly enriched in two categories, i.e., biological process and molecular function. Two group IIc members (LchiWRKY10 and LchiWRKY37) contain unique WRKY element sequence variants (WRKYGKK and WRKYGKS). Gene structure analysis showed that most *LchiWRKYs* possess 3 exons and two different types of introns: the R- and V-type which are both contained within the WRKY domain (WD). Additional promoter cis-element analysis indicated that 12 cis-elements that play different functions in environmental adaptability occur across all *LchiWRKY* groups. Heat, cold, and drought stress mainly induced the expression of group II and I *LchiWRKYs*, some of which had undergone gene duplication during evolution, and more than half of which had three exons. *LchiWRKY33* mainly responded to cold stress and *LchiWRKY25* mainly responded to heat stress, and *LchiWRKY18* mainly responded to drought stress, which was almost 4-fold highly expressed, while 5 *LchiWRKYs* (*LchiWRKY5*, *LchiWRKY23*, *LchiWRKY14*, *LchiWRKY27,* and *LchiWRKY36*) responded equally three stresses with more than 6-fold expression. Subcellular localization analysis showed that all *LchiWRKYs* were localized in the nucleus, and subcellular localization experiments of *LchiWRKY18* and *36* also showed that these two transcription factors were expressed in the nucleus.

**Conclusions:**

This study shows that in *Liriodendron chinense*, several *WRKY* genes like *LchiWRKY33*, *LchiWRKY25,* and *LchiWRKY18*, respond to cold or heat or drought stress, suggesting that they may indeed play a role in regulating the tree’s response to such conditions. This information will prove a pivotal role in directing further studies on the function of the *LchiWRKY* gene family in abiotic stress response and provides a theoretical basis for popularizing afforestation in different regions of China.

**Supplementary Information:**

The online version contains supplementary material available at 10.1186/s12870-021-03371-1.

## Background

Transcription factors (TFs) are important proteins that bind to specific DNA motifs to regulate gene expression and play crucial roles in plant growth, development, metabolism, and stress response [1, 2]. WRKY transcription factors were first identified in plants and form one of their largest gene families [3]. After the first *WRKY* gene was cloned in sweet potato, the *WRKY* gene family function has been studied in several model plants, such as *Arabidopsis thaliana* (72), *Oryza sativa* (76), *Amborella trichopoda* (31), and *Solanum lycopersicum* (81) [4, 5]. The main defining feature of WRKY proteins is their WRKY domain, which contains a conserved amino acid sequence WRKYGQK at the N-terminus and a C2H2 or C2HC zinc motif at the C-terminus of about 60 amino acids in length [1, 6]. Both the heptapeptide sequence and zinc-finger motif are required for the high binding affinity of WRKY TFs to the consensus W-box (TTGACC/T) cis-elements in their target genes, which regulates target gene expression [7, 8]. Based on the number of WRKY domains and the structure of their zinc-finger motifs, WRKY proteins can be classified into three main groups: groups I, II, and III. Group II is specified by WRKY proteins containing a single WRKY domain with a C2H2 motif and can be further divided into five subgroups: IIa, IIb, IIc, IId, and IIe. Group I contains WRKY proteins with two WRKY domains including a C2H2 motif. The remaining WRKY proteins, containing a WRKY domain along with a C2HC motif, belong to group III [9, 10].

The evolution of the *WRKY* gene family has been studied in a large number of reports, but a clear evolutionary history has so far not yet been defined. This has resulted in an active debate on the origin of individual *WRKY* genes and gene groups in different plant species [3, 11]. According to a large phylogenetic tree using higher plants, the *WRKY* gene family can be classified into four clades, including groups I + IIc, groups IIa + IIb, group IId, and group IIe [3, 12]. Previous reports have suggested two alternative hypotheses for the evolution of the *WRKY* family: The “Group I Hypothesis” and the “IIa + b Separate Hypothesis”. The “Group I Hypothesis” postulates that the C-terminal WRKY domains of group I genes are the most ancient and that all other *WRKY* genes are derived from these, whereas the “IIa + b Separate Hypothesis” suggests that groups IIa and IIb evolved directly from a single domain algal gene, independent from the group I-derived lineage [3, 13, 14].

In addition, WRKY transcription factors are involved in a plant’s stress response to herbivores, pathogens, and nematodes [15, 16]. Substantial evidence has shown that the WRKY TFs play significant roles in the signaling and regulation of gene expression during biotic and abiotic stress responses [17, 18]. For example, In Camelina (*Camelina sativa* (L.) Crantz), *CsWRKY21* was highly expressed under cold stress and *CsWRKY22* was highly expressed under drought stress [17]. In the Grape (*Vitis vinifera* L.), *VvWRKY11* is involved in the response to dehydration stress, and *VvWRKY24* expression is induced by cold stress [18]. In Cucumber (*Cucumis sativus* L.), five *CsWRKYs* responded strongly to salt stress and high-temperature stress [4]. In Wheat (*Triticum aestivum*), the expression of *TaWRKY75-A* was highly induced under salt stress [19]. In *Ipomoea trifida* (H.B.K.) G. Don, 11 *ItfWRKYs* were highly expressed under four abiotic stresses: cold, heat, salt, and drought [16]. In *Arabidopsis*, *AtWRKY39* positively regulates the cooperation between the SA and JA activated signaling pathways that mediate the response to heat stress, while *AtWRKY8* is highly expressed in plant roots and significantly upregulated under salt stress [20, 21].

*Liriodendron*, a basal angiosperm genus, resides within the Magnoliaceae family and has diverged into two distinct species: the East Asian (*L. chinense* (*Hemsley*) *Sargent*) and the North-East American (*L. tulipifera Linn*) [22, 23]. *L. chinense* is mainly distributed in the south of the Yangtze River, which has further diverged into two separate lineages, the eastern and western groups in China [23]. The flowering mutant gene *super long blooming 1*(*slb1*) of *L. chinense* was identified by transcriptome analysis [24]. There are few reports on abiotic stress research, such as auxin efflux carrier *PIN*-*FORMED* (*PIN*) [25], gibberellin oxidase (*GAox*) [26], myeloblastosis (*MYB*) [27], and C-regeneration binding factors (*CBF*) gene family [28]. *Liriodendron hybrids* are intraspecific hybrids crossed by *L. chinense* and *L. tulipfera,* which are valuable ornamental tree species in both private and public spaces because of their fast growth, drought tolerance, and strong resistance to diseases and insect pests [29, 30]. Under aluminum (Al) stress, glutamate decarboxylase (GAD) was up-regulated in *Liriodendron hybrids*, and enhanced the Al tolerance [31]. Adverse temperature and soil conditions can inhibit the growth of *L. chinense*, such as cold, heat, and drought stress. Knowledge is still lacking on how *L. chinense* deals with abiotic stress at a molecular level. *WRKY* family is involved in plant growth and development and abiotic stress response and has been reported in many species with available genome sequences. The recently published *L. chinense* genome now provides ample resources with which to carry out bioinformatics-based identification and analysis of WRKY TFs.

In this study, we identified 44 *LchiWRKY* genes and classified them into three main groups. A comprehensive analysis, including determination of exon-intron structure, motif, gene ontology, gene duplication events, chromosome distribution, subcellular localization, and comparative phylogenetic analysis was performed. Furthermore, global transcriptome expression analysis was performed to identify the gene expression pattern of specific *WRKY* gene family members under different abiotic stress conditions in *L. chinense*. In addition, Quantitative real-time PCR (qRT-PCR) was used to verify the expression levels of *LchiWRKY* genes with different expression patterns. Subcellular localization experiment verified the expression position of LchiWRKY transcription factors. This study provides valuable clues for the functional characterization of the *WRKY* gene family in *L. chinense*.

This research reveals the functional roles of the *L. chinenese WRKY* genes under abiotic stress and provides a valuable theoretical basis for increasing forest yield and promoting afforestation of Liriodendron.

## Results

### The *L. chinense* genome contains a small number of *WRKY* genes that vary greatly in length

Since *WRKY* genes have previously been demonstrated to be involved in abiotic stress response, we set out to characterize the *L. chinense WRKY* gene family. To gain an understanding of the gene diversity present within this family, we determined the basic physicochemical properties of the WRKY protein products, including sequence length, molecular weight (MW), isoelectric point (pI) and other indexes for all 44 identified LchiWRKY (Additional file 1: Table S1). The size of the LchiWRKY proteins varied dramatically, with LchiWRKY37 being the shortest protein at 143 amino acids (aa) in length, whereas LchiWRKY42, the longest, contains 787 aa. Their MW ranges from 16.274 to 90.094 kDa, and the pI from 4.95 (LchiWRKY41) to 9.89 (LchiWRKY36). These data suggest that different LchiWRKY proteins might operate in various microenvironments. The values of the grand average of hydropathicity were negative for all LchiWRKY proteins, indicating that they are all hydrophilic proteins. Almost all LchiWRKYs were defined as unstable proteins, and only 3 LchiWRKYs (LchiWRKY35, LchiWRKY37, LchiWRKY40) with an instability index of less than 40 were considered to be stable proteins.

The number of *WRKY* gene family in *L. chinense* is small, and their physical and chemical characteristics vary widely, so it is necessary to explore their taxonomic and phylogenetic characteristics.

### The *LchiWRKY* gene family consists of three distinct phylogenetic groups

**Fig. 1:**
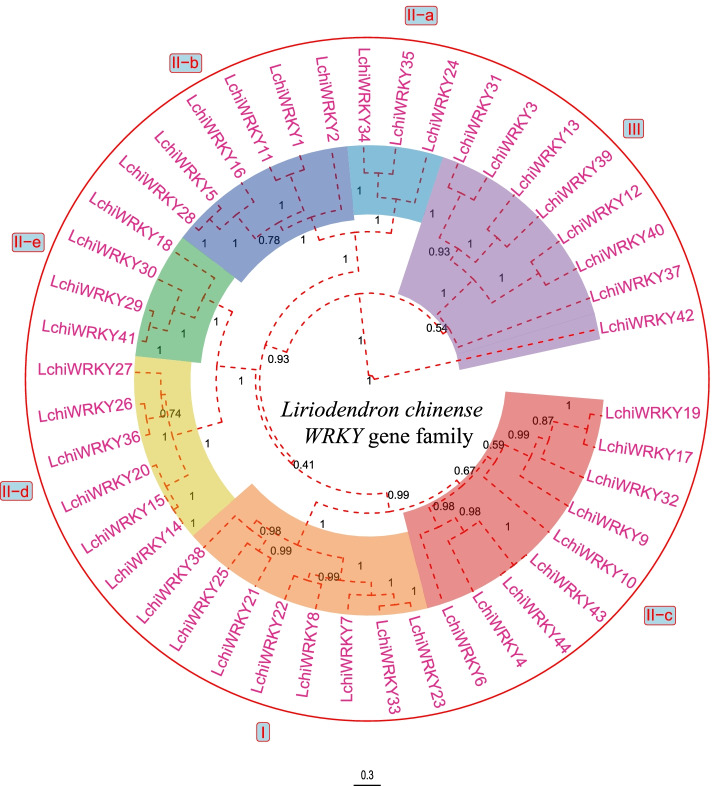
Phylogenetic tree of LchiWRKYs. A total of 44 LchiWRKY proteins were aligned using MUSCLE (v3.8.31), and a phylogenetic tree was constructed using BEAST (v2.6.6) set to the Bayesian method with 10,000,000 MCMC chain length. Each color indicates an individual closely related group of WRKY proteins (I-III) within the phylogenetic tree, Posterior probability limit was 1.0 and the posterior value was shown on the branch. The scale bar indicates the branch length of the phylogenetic tree. The posterior value below 0.5 indicates a low homology among the different LchiWRKY protein sequences

We constructed a phylogenetic tree that would indicate the LchiWRKY protein family’s evolutionary interrelatedness. We based our phylogenetic tree on a multiple sequence alignment of the WRKY full length of each protein. We found that the 44 *LchiWRKYs* are divided into three separate phylogenetic groups: Group I, Group II, and Group III [13], out of which Group II contains five subgroups, being Group IIa, Group IIb, Group IIc, Group IId, and Group IIe (Fig. 1 and Additional file 2: Table S2). The largest subgroup is IIc with 9 members, and the smallest subgroup is IIa with 4 members.

**Fig. 2:**
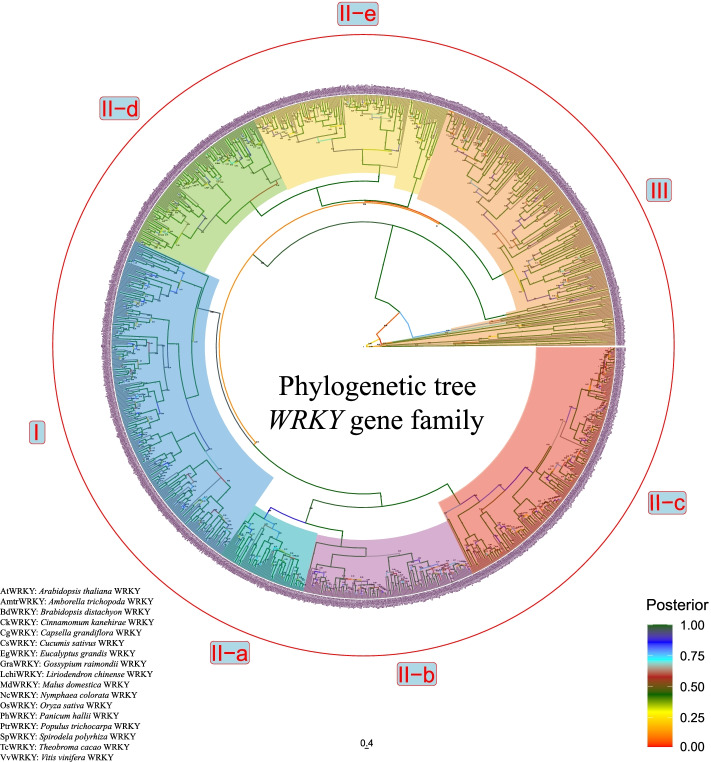
Unrooted Classification tree representing relationships among *WRKY* genes of 17 species. A total of 1308 WRKY proteins were aligned by MUSCLE(v3.8.31), and a phylogenetic tree was constructed using BEAST (v2.6.6) set to the Bayesian method with 10,000,000 MCMC chain length. Each label indicates an individual phylogenetic group (I-III). The posterior probability limit was 1.0 and the posterior value was shown on the branch. Different highlighted colors of clades represent the different groups and different branch colors represent the corresponding posterior values. The scale bar indicates the branch length of the phylogenetic tree. The legend indicated the posterior values, which below 0.5 indicate a low homology among the protein sequences involved

To see how the *L. chinense WRKY* gene family would compare to model plants that each represent a different evolutionary branch, we constructed a phylogenetic tree including the WRKY protein sequences from basal angiosperms, magnolias, monocotyledons, dicotyledons. There are 17 species, including the basal angiosperm *Amborella trichopoda, Nymphaea colorata*, magnolia *Cinnamomum kanehirae, Liriodendron chinense*, monocotyledons (*Brachypodium distachyon*, *Oryza sativa, Panicum hallii, Spirodela polyrhiza*), dicotyledons (*Arabidopsis thaliana*, *Capsella grandiflora*, *Cucumis sativus*, *Eucalyptus grandis*, *Malus domestica*, *Gossypium raimondii*, *Populus trichocarpa*, *Theobroma cacao*, *Vitis vinifera*). *A. trichopoda* is a basal angiosperm, having diverged earlier than *L. chinense*, the others are di- and monocotyledonous plants respectively that have diverged significantly later than *L. chinense* [23]. A total of 1308 WRKY proteins were used to construct a Bayesian phylogenetic tree as described in the Methods section (Fig. 2 and Additional file 3: Table S3).

All 1308 WRKYs clustered across major clades of the classification group. We frequently found WRKY members from all 17 species that belong to one specific group (I, II, III) to be clustering to the same clade. We found LchiWRKY protein sequences to be more closely related to their homologs from *C. kanehirae* than those from monocotyledons and dicotyledons. This finding is consistent with the evolutionary status of *L. chinense* being closer to *A. trichopoda*, another basal angiosperm, than model plants *A. thaliana* and *O. sativa*, two more recently evolved dicotyledonous and monocotyledons species [1].

### The *LchiWRKY* family was at a relatively primitive evolutionary status and had fewer family members

Interestingly, the total number of *WRKY* families in the majority of monocotyledons and dicotyledons is more than that in *L. chinense* (44) and *A. trichopoda* (31) (Table 1 and Additional file 4: Table S4), for example, among monocotyledons, *S.polyrhiza* has the least number of *WRKY* families (44), and among dicotyledons, *V. vinifera* has the least number of *WRKY* families (60), but most of them were more than that of *L. chinense*. For Magnolia plants, two whole-genome duplication (WGD) events occurred during the evolution of *C. kanehirae* [32], and only one WGD event occurred in *L. chinense* [23], which may lead to a greater number of *WRKY* genes in *C. kanehirae (73)* compared to *L. chinense (44)*. As the three subgroups (I, II, II) of plants of different evolutionary statuses are clustered into the same branch, so it is speculated that the *WRKY* family is relatively conservative in the process of evolution. We infer that different plant subgroups have different numbers due to differential expansion speeds and finally lead to different total family numbers, as a result, the *WRKY* family number of some basal angiosperms and magnolias expanded slower than monocotyledons and dicotyledons [14].

**Table 1:** The total number and subgroups of *WRKY* families in 17 species

Taxon	Species	Total_number	Group I	Group IIa	Group IIb	Group IIc	Group IId	Group IIe	Group III
Basal Angiosperms	*A.trichopoda*	31	7	2	4	9	3	3	3
Basal Angiosperms	*N.colorata*	65	18	6	9	10	7	10	5
Magnolia	*L.chinense*	44	7	3	6	8	4	7	9
Magnolia	*C.kanehirae*	73	15	6	8	12	9	12	11
Monocotyledon	*S.polyrhiza*	44	6	2	6	11	6	8	5
Monocotyledon	*O.sativa*	76	16	4	8	10	10	5	23
Monocotyledon	*B.distachyon*	89	17	5	5	16	10	10	26
Monocotyledon	*P.hallii*	120	35	7	16	18	13	17	14
Dicotyledon	*V.vinifera*	60	16	4	8	12	6	8	6
Dicotyledon	*T.cacao*	61	14	3	8	10	6	7	13
Dicotyledon	*C.sativus*	62	18	4	5	11	8	8	8
Dicotyledon	*C.grandiflora*	69	16	3	8	13	6	8	15
Dicotyledon	*A.thaliana*	72	18	3	8	12	7	8	16
Dicotyledon	*E.grandis*	79	17	7	10	17	8	7	13
Dicotyledon	*P.trichocarpa*	102	16	4	8	13	12	11	38
Dicotyledon	*G.raimondii*	102	25	5	9	22	14	16	11
Dicotyledon	*M.domestica*	159	32	8	17	29	14	21	38

Total_number indicates the total number of *WRKY* families, Group I ~ III indicates the subgroups number of *WRKY* families

**Fig. 3:**
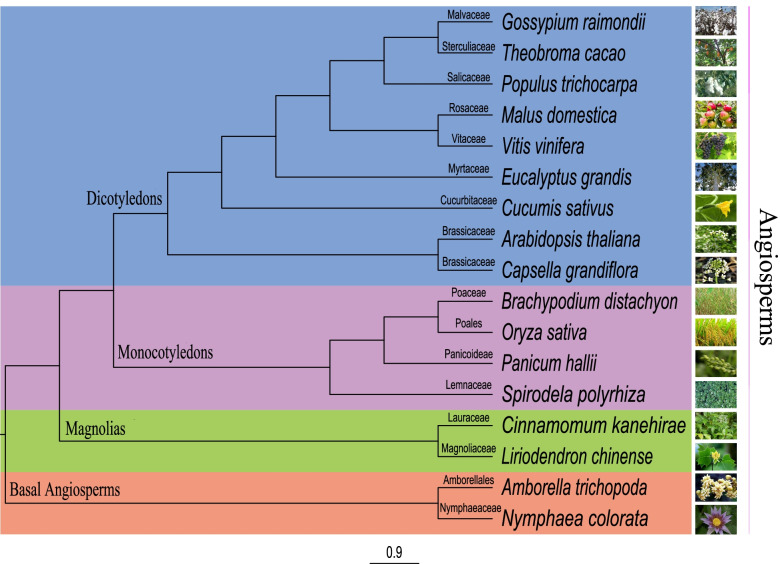
Rooted phylogenetic tree of the 17 species *WRKY* families. A total of 1308 WRKY proteins of 17 species were constructed the rooted phylogenetic tree by OrthoFinder (v2.2.3), the blue color represented dicotyledons and violet color represented monocotyledons, the green color represented magnolias, and the red color was represented basal angiosperms. The scale bar indicates the branch length of the phylogenetic tree. The picture on the right panel was the picture corresponding to each species, and the name of the family of each species was annotated on the corresponding branch of the evolutionary tree

Then we used the OrthoFinder (v2.2.3) to explore the *WRKY* evolutionary status of all the 17 species (Fig. 3 and Additional file 4: Table S4), and the phylogenetic analysis showed that the evolutionary status of *WRKY* of *L. chinense* was closer to the Magnolia *C. kanehirae*, and the evolutionary status of both species is relatively lower than that of higher angiosperms, including monocotyledons and dicotyledons, while higher than basal angiosperms *A. trichopoda* and *N.colorata* (Fig. 3). These results suggested that *L. chinense* might experience a slower expansion model during evolution, resulting in a smaller number of family members than other higher plants. So it is necessary to explore the expansion pattern of the *LchiWRKY* gene family.

### The *LchiWRKY* gene family expanded due to gene tandem duplication events in the evolutionary past

**Fig. 4:**
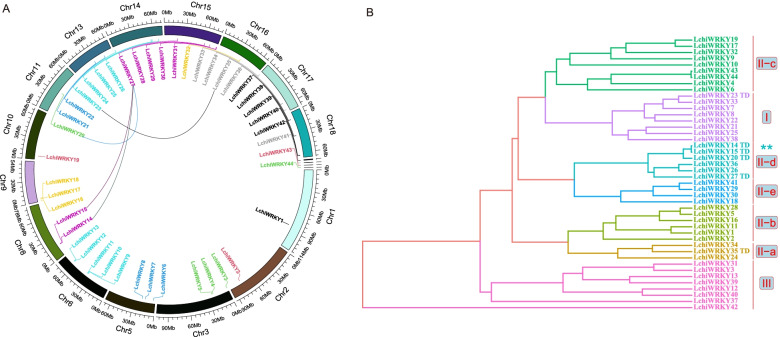
*LchiWRKY* chromosomal localization and tandem duplication events in the *L. chinense* genome. **A** The chromosomal distribution and positioning of *LchiWRKYs* are plotted using a Circos plot. The 15 chromosomes that contain *WRKY* genes (each indicated by a different color) are plotted along with an Mb (million base pair) scale (1 tick = 10 Mb). Individual *LchiWRKY* gene positions are labeled using the *WRKY* gene name. Where individual chromosomes (bars) are labeled with respective *LchiWRKY* genes in the circle, each colored curve indicates the gene duplication event of *LchiWRKYs* across the chromosomes. **B** Tandem duplication events distributed across the phylogenetic tree groups. TD indicates a tandem duplication event, each group was labeled with differently colored branches, ** represent the most obvious group of TD event

To understand how the identified *LchiWRKY* genes are distributed over the *L. chinense* chromosomal landscape, we mapped their sequences onto its 19 chromosomes [23] (Fig. 4 and Additional files 5 and 6: Tables S5–S6). Considering the high diversity with the *L. chinense WRKY* gene family, we asked how then the different genes would be interrelated. Using *L. chinense* genome annotation data, we identified a total of 44 *LchiWRKYs*, which were renamed from *LchiWRKY1* to *LchiWRKY40* based on their chromosomal positioning. An additional four genes (*LchiWRKY41*, *LchiWRKY42*, *LchiWRKY43*, and *LchiWRKY44*) that were mapped to yet unassembled scaffolds were named *LchiWRKY41* ~ *LchiWRKY 44* respectively.

We found 40 *LchiWRKYs* to be unevenly distributed across 15 out of the 19 *L. chinense* chromosomes, with chromosomes 4, 7, 12, and 19 devoid of any *WRKY* gene. However, the remaining 4 genes mapped onto four separate scaffolds were not yet assembled into full chromosomes. Overall, the number of *WRKY* genes per chromosome varied from 0 to 5, with no apparent correlation between chromosomal length and the number of *WRKY* genes present (Fig. 4A).

Our previous study mapped several gene duplication events that occurred in the evolutionary past of *L. chinense* [23]. Such events could have also affected the number of *WRKY* genes in the *L. chinense* genome, as well as their distribution over the genome [3]. Duplication events are known to occur at a regional scale via tandem duplication, copying one gene’s sequence in the direct vicinity of the original (< 200 kb), but may also occur at a larger scale via segmental duplication, copying whole sections of chromosomes [3, 13]. The former is often caused by DNA replication errors, whereas the latter may result from polyploidy events followed by chromosomal rearrangements [3, 33].

We could find a total of 4 *WRKY* gene pairs on the *L. chinense* genome. Out of these, no gene resulted from a segmental duplication event (Fig. 4A). The 4 events (*LchiWRKY27*-*LchiWRKY15*, *LchiWRKY27-LchiWRKY14*, *LchiWRKY27-LchiWRKY20*, *LchiWRKY23-LchiWRKY35*) have all likely resulted from tandem duplication, leading to 6 additional *WRKY* genes (*LchiWRKY14*,*15*,*20*,*23*,*27*, and *35*) in the *L. chinense* genome, which distributed on Chr8, 11,14,15, and 16. Two of them were in group I (*LchiWRKY23*) and IIa (*LchiWRKY35*), the others were in group IId (Fig. 4B). These results indicate that a few gene duplication events like group IId have indeed contributed to the expansion of the *LchiWRKY* gene family in the evolutionary past.

To gain an understanding of whether or not the duplicated *LchiWRKY* genes contribute to organism fitness, we measured the Ka/Ks nucleotide substitution ratios of 4 individual gene pairs to study the exerted selective pressure (Table 2).

**Table 2:** Ka/Ks values of *LchiWRKY* gene pairs

Sequence	Method	Ka	Ks	Ka/Ks
LchiWRKY27&LchiWRKY15	MA	0.472679	0.988463	0.478197
LchiWRKY27&LchiWRKY14	MA	0.472679	0.988463	0.478197
LchiWRKY27&LchiWRKY20	MA	0.472679	0.988463	0.478197
LchiWRKY23&LchiWRKY35	MA	0.994488	1.10966	0.896212

The sequences represent duplicated gene pairs, MA represents a model that averages parameters across 14 candidate models, Ka, Ks, Ka/Ks represent the values of Ka (non-synonymous substitutions per non-synonymous site), Ks (synonymous substitutions per synonymous site), and Ka/Ks respectively

Generally, a Ka/Ks ratio > 1 is consistent with positive selection, while Ka/Ks < 1 indicates a purifying or negative selection [34]. We found their Ka/Ks ratios all to be < 1, indicating that the duplicated *LchiWRKYs* have undergone a synonymous substitution or purifying selection with limited functional divergence during their evolutionary history.

The analysis of gene replication events and selection pressure showed that the *WRKY* family expanded mainly through tandem duplications, but they were subjected to purifying selection in the process of evolution, so it is necessary to further explore the structural characteristics and functions of *LchiWRKYs*.

### The *LchiWRKYs* contained multiple motifs and gene ontology enrichment analysis

**Fig. 5:**
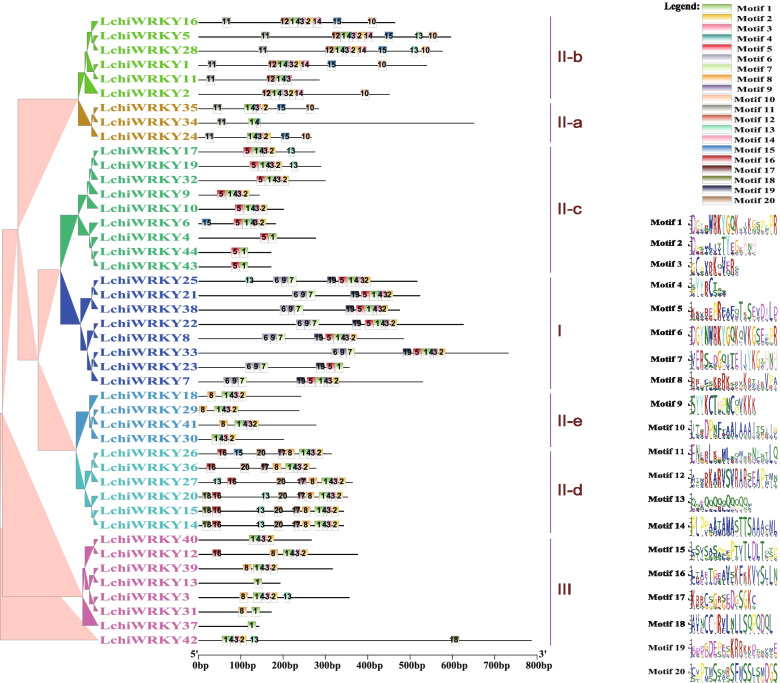
LchiWRKY motifs structure. Left panel: an unrooted phylogenetic tree constructed using Beast (v2.6.6) set to the Bayesian method (left) and distribution of conserved motifs in the LchiWRKY proteins (right, scale = aa length), each group was labeled and with the different color branch, the differently colored boxes represent different motifs and their position in each WRKY protein sequence, the number represents the specific motif and component (see legend)

To further know the structure of *LchiWRKYs* and their phylogenetic relationships, we performed a gene sequence analysis for conserved motifs using the MEME suite software package [35], which identified 20 different conserved sequence motifs (motif 1–20) in the LchiWRKY protein family (Fig. 5 and Additional file 7: Table S7).

We found a single motif (motif 1, a WRKY motif) to be widely distributed among all members of the *LchiWRKY* family, while all others were specific to one or a few phylogenetic groups we defined previously (Fig. 5). For example, motif 6 (a WRKY motif) and motifs 7, 9, and 19 are only present in Group I members (*LchiWRKY38*, *25*, *21*, *22*, *8*, *7*, *33*, and *23*), motif 10 and 11 are specific to Groups IIa (*LchiWRKY34*, *35*, and *24*) and IIb (*LchiWRKY16*, *5*, *28*, *1*, *11*, and *2*), motif 12 to Group IIb, motif 5 to Group IIc (*LchiWRKY17, 19, 32, 9, 10, 6,* *4*, *44* and *43*) and I and motif 16, 17 and 20 to Group IId (*LchiWRKY26, 36, 27, 20, 15* and *14*). Remarkably, Groups IIe (*LchiWRKY18, 29, 41 and 30*) and III (*LchiWRKY40, 12, 39, 13, 3, 31, 37* and *42*) did not contain any conserved sequence motifs, suggesting an increased sequence diversification of these groups (Fig. 5). According to most reports, the functions of some motifs remain to be elucidated. Overall, the closely related *LchiWRKYs* in the phylogenetic clades shared similar motif compositions, suggesting that the *LchiWRKYs* within the same phylogenetic group may have similar functions.

To further understand the function of the identified 44 *WRKY* genes, we performed gene ontology (GO) annotation analysis (Additional file 8: Fig. S1). The results showed that a total of 34 genes were significantly enriched in different GO terms, mainly including 2 categories and 10 subcategories. In the molecular function category, 34 genes were significantly enriched in 3 subcategories, including ‘Sequence−specific DNA binding’ (GO: 0043565), ‘DNA binding transcription factor activity’ (GO: 0003700) and ‘Transcription regulator activity’ (GO: 0140110). In the biological process category, they were mainly enriched in 7 subcategories, including ‘Regulation of transcription, DNA-templated (GO: 0006355), ‘Regulation of nucleic acid−templated transcription’ (GO: 1903506), ‘Regulation of RNA biosynthetic process’ (GO: 2001141), ‘Regulation of RNA metabolic process’ (GO: 0051252), ‘Regulation of biosynthetic process’ (GO: 0009889), ‘Regulation of macromolecule biosynthetic process’ (GO: 0010556) and ‘Regulation of cellular biosynthetic process’ (GO: 0031326). Molecular functional enrichment is more significant than others, including three terms: DNA binding transcription factor activity, sequence-specific DNA binding, and transcription regulator activity, indicating that these three terms may be the main functions of *WRKY* genes. The three molecular functional subcategories are more representative than the subcategories of biological processes.

### Diversity of gene structure and two main promoters cis-element regulator analysis

**Fig. 6:**
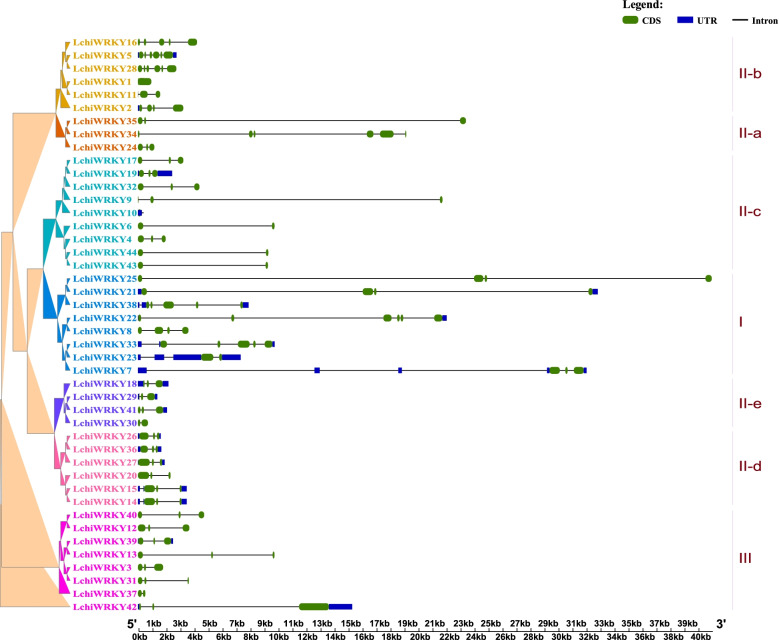
Exon-intron structure of *LchiWRKYs*. An unrooted phylogenetic tree constructed using Beast (v2.6.6) set to the Bayesian method (left), green boxes indicate exons; blue boxes indicate untranslated 5′- and 3′-regions; black lines indicate introns, scale = bp indicates sequence length, each group was labeled with a differently colored branch

We next examined gene structure by studying the exon-intron organization and cis-element in all identified *LchiWRKY* genes, to gain more insight into the evolution of the *WRKY* family in *L. chinense*. Firstly, we could observe a large variation in gene size of different *LchiWRKYs*, ranging from 525 bp (*LchiWRKY37*) to 40,981 bp (*LchiWRKY25*) (Additional file 9: Table S8 and Fig. 6).

This suggested a large variation in intron length since we had seen previously that the different *LchiWRKY* CDS sequences were of a much more similar size. The exon number varied between 2 and 6 exons for all *LchiWRKY* genes, with the majority of genes (27 / 61.36%) having a total of 3 exons (Additional file 9: Table S8 and Fig. 6). Two types of introns exist in the conserved WDs. One of the introns is spliced exactly at the R residue, We classified this phase-2 intron as an R-type intron. The other is localized before the V residue, which is at the sixth amino acid after the second C residue in the C2H2 motif in the zinc finger region. This phase-0 intron is designated as a V-type intron. We could identify two major types of introns within the conserved WRKY domains of the *LchiWRKY* genes (Additional file 10: Fig. S2), which are similar to those conserved in *Arabidopsis* [13]. Interestingly, phylogenetic groups IIa and IIb possess a V-type, or phase-0 intron, while all other groups (I, IIc-e, and III) contain an R-type, or phase-2 intron (excepting the *LchiWRKY42* gene).

The WRKY domain sequence within the *L. chinense WRKY* gene family shows a high degree of conservation (Additional file 11: Table S9), with only two WRKY sequence variants (WRKYGKK and WRKYGKS) present in the LchiWRKY10 and LchiWRKY37 proteins (both in subgroup II-c) respectively. Six proteins, classified in groups II-c and III, lack a C-terminal zinc-motif. Group I contains 8 LchiWRKYs, with each possessing two WDs and C2H2-type zinc finger motifs (C-X4-C-X22-H-X1-H) (Additional file 11: Table S9). Group II is comprised of 29 proteins, each containing a single WD and C2H2-type zinc finger structure (C-X4–5-C-X23-H-X1-H) (Additional file 11: Table S9). We further divided Group II into five subgroups according to their clusters in the phylogenetic analyses, including IIa, IIb, IIc, IId, and IIe with 3, 6, 10, 6, and 4 members, respectively. Finally, 7 LchiWRKYs with a single WD were assigned to Group III, because of their unique C2HC zinc-finger structure (C-X7-C-X23-H-X1-C).

**Fig. 7:**
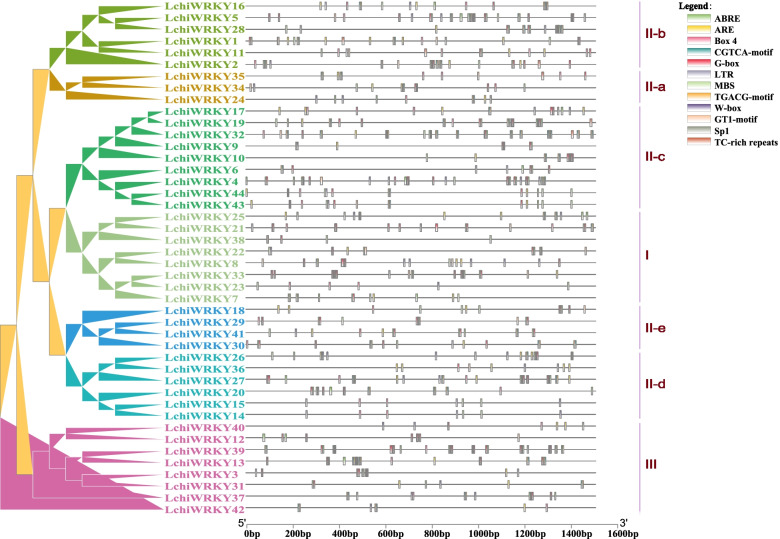
Cis-acting elements of *LchiWRKYs*. An unrooted phylogenetic tree is shown, constructed using BEAST (v2.6.6) set to the Bayesian method and 11 cis-acting elements on the 1.5 kb of *LchiWRKYs* promoter sequences. The differently colored boxes represent different cis-acting elements, each group was labeled with a differently colored branch

We then used the PlantCARE website to analyze 1.5 kb of the 5′ upstream promoter regions of the *LchiWRKY* genes to identify any conserved cis-acting elements [4]. Various types of cis-acting elements were found and all *LchiWRKY* genes contained several cis-acting elements in their promoter regions. The 12 most common elements are summarized in Fig. 7 (Additional file 12: Table S10). There are two types of cis-elements. One is related to growth and development, including WRKY TF binding element (W-box) and four light-responsive elements (Sp1, Box 4, G-box, and GT1 motif). The other is related to stress response, including abscisic acid-responsiveness (ABRE), MeJA-responsiveness (CGTCA and TGACG motifs), anaerobic element (ARE), drought stress-responsive element (MBS), defense and stress-responsive element (TC-rich), low-temperature stress responsive element (LTR). Several of the *cis*-elements we identified showed a specific distribution across the different *L. chinense WRKY* gene groups. For example, group II-a lacks any LTR elements, suggesting that this group may not be sensitive to low-temperature stress. The Sp1 element can be found in all groups except IId and IIe, showing that the genes in these two groups might not be as responsive to light. Moreover, the TC-rich element could only be detected in groups IIa, IIb, and IIc, indicating that these three groups might be mainly involved in defense and stress response. Finally, the MBS element is not present in group III genes, implying that these genes may not be induced under drought stress. In summary, the *L. chinense WRKY* gene groups seem to possess their own sets of *cis*-responsive elements, indicating that the different groups may have specialized towards responding to specific sets of stimuli.

Analysis of gene structure, motif and cis-regulatory elements showed that there were two WRKY structural variants and intron insertion structures, 2 GO categories and 12 *cis*-elements in the *WRKY* family, which regulated growth and development and abiotic stress response. Therefore, the expression profile of *WRKY* in growth and abiotic stress needs to be further studied.

### The *LchiWRKY* group II responds strongly to multiple abiotic stresses

**Fig. 8:**
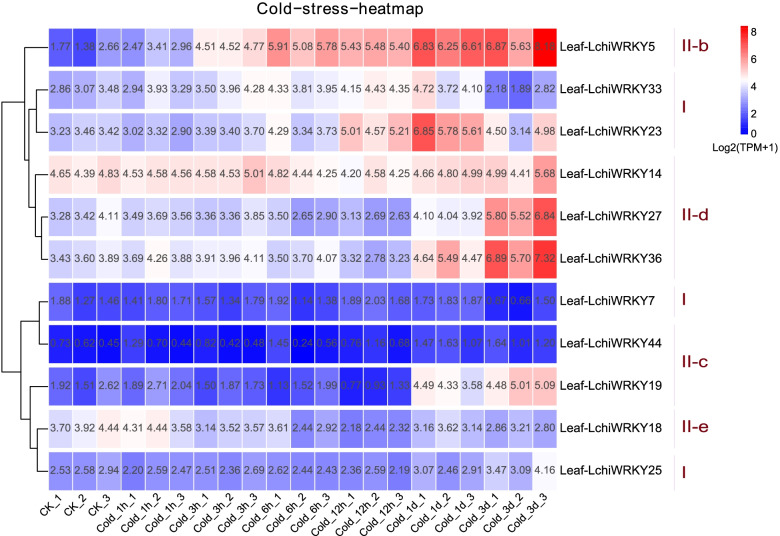
*LchiWRKY* expression patterns in response to cold stress. Expression levels of *LchiWRKYs* at successive time points in response to cold stress. CK_1, CK_2, CK_3 represent three biological repetitions of the control group, Cold_1h, Cold_3h, Cold_6h, Cold_12h, Cold_1d, Cold_3d represent three biological repetitions of each time point (1 h, 3 h, 6 h, 12 h, 1d, 3d), Leaf-LchiWRKYs represent *LchiWRKY* gene of leaf tissue. The transcript abundance level was normalized and hierarchically clustered by using log2(TPM + 1) values. Colored blocks indicate decreased (blue) or increased (red) transcript accumulation among the analyzed tissues. The heat map was generated by the R package pheatmap (v1.0.12)

**Fig. 9:**
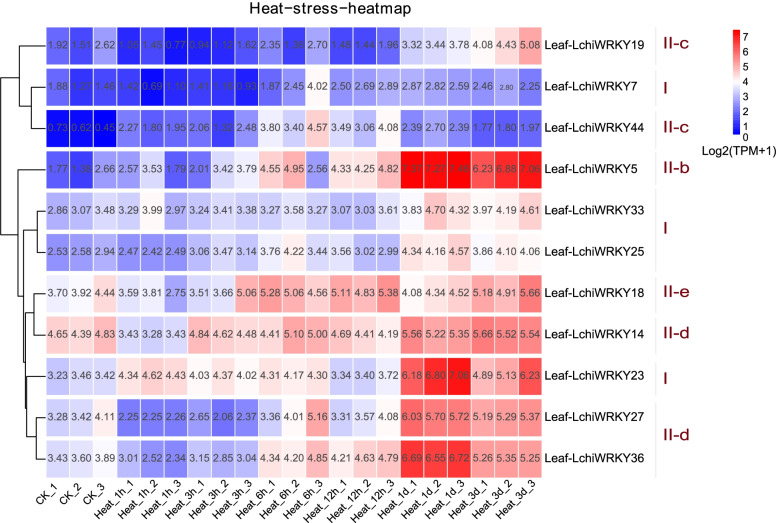
*LchiWRKY* expression patterns in response to heat stress. Expression levels of *LchiWRKYs* at successive time points in response to heat stress. CK_1,CK_2,CK_3 represent three biological repetitions of the control group, Heat_1h, Heat_3h, Heat_6h, Heat_12h, Heat_1d, Heat_3d represent three biological repetitions of each time point (1 h, 3 h, 6 h, 12 h, 1d, 3d), Leaf-LchiWRKYs represent *LchiWRKY* gene of leaf tissue. The transcript abundance level was normalized and hierarchically clustered by using log2(TPM + 1) values. Colored blocks indicate decreased (blue) or increased (red) transcript accumulation among the analyzed tissues. The heat map was generated by the R package pheatmap (v1.0.12)

**Fig. 10:**
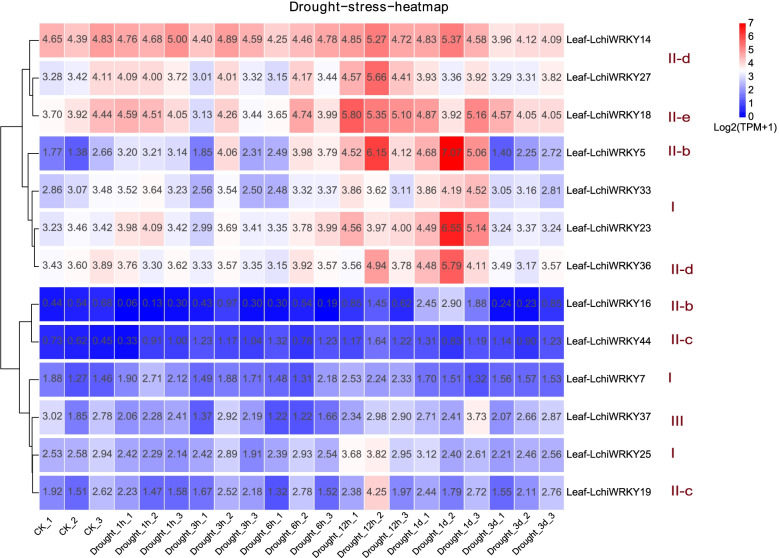
*LchiWRKY* expression patterns in response to drought stress. Expression levels of *LchiWRKYs* at successive time points in response to drought stress. CK_1,CK_2,CK_3 represent three biological repetitions of the control group, Dought_1h, Dought_3h, Dought_6h, Dought_12h, Dought_1d, Dought_3d represent three biological repetitions of each time point (1 h, 3 h, 6 h, 12 h, 1d, 3d), Leaf-LchiWRKYs represent *LchiWRKY* gene of leaf tissue. The transcript abundance level was normalized and hierarchically clustered by using log2(TPM + 1) values. Colored blocks indicate decreased (blue) or increased (red) transcript accumulation among the analyzed tissues. The heat map was generated by the R package pheatmap (v1.0.12)

Environmental stress can affect a plant’s health and growth, as well as influence the regulation of crucial molecular processes. Under adverse conditions, a large number of stress-related genes are induced to help plants deal with stress. Therefore, to understand how *L. chinense* deals with abiotic stress and see whether *WRKY* genes play a role in stress response, as well as their regulatory patterns, we used leaf tissue transcriptome data from 7 subsequent time points (0 h, 1 h, 3 h, 6 h, 12 h, 1d, and 3d) to explore the expression of *LchiWRKY* genes in response to cold, heat and drought stress (Figs. 8, 9 and 10).

A total of 11 genes were induced by cold stress (Fig. 8 and Additional file 13: Table S11), their expression patterns were clustered into two groups, including persistent low expression and high expression at different time points. *LchiWRKY5*, *23*, *14*, *27*, *36*, *19* showed peak expression at the 1d and 3d time points, while downregulated at the other time points. Only two genes showed long-lasting and continuous up-regulation after cold stress, with *LchiWRKY5* being up-regulated from 3 h to 3d and *LchiWRKY33* from 3 h to 1d, *LchiWRKY14* was highly expressed at almost all time points. These findings may suggest that *LchiWRKY5* and *LchiWRKY14* mainly promote *L. chinense* in response to low-temperature stress. *LchiWRKY* family may play a major regulatory role at 1d and 3d response time points.

The response of *LchiWRKY* to high-temperature stress was more obvious than that to low-temperature stress, (Fig. 9 and Additional file 14: Table S12). A total of 11 genes were induced by heat stress, and the expression patterns were clustered into two groups, including low expression patterns at almost all time points and high expression patterns at continuous time points (3 h ~ 3d). *LchiWRKY7* were lowly expressed in all-time points, most of them were differentially expressed in all-time points. *LchiWRKY5, 14, 23, 27, 36* were up-regulated significantly at the 1d and 3d time points, similar to how they responded to cold stress. *LchiWRKY44, 18* reached its expression peak at 6 h and 12 h time points, while *LchiWRKY33*, *25* were up-regulated at the 1d and 3d time points, which were different from cold stress. Compared with cold stress, *LchiWRKY18, 25* was specifically high expressed from 1d to 3d under heat stress. Under heat stress, we speculate that 1d and 3d may be important response time points.

Under drought stress (Fig. 10 and Additional file 15: Table S13), a total of 13 *LchiWRKY* family members responded, and their expression patterns were divided into two groups, one group was a high expression from 6 h to 1 day, the other group was a low expression at all time points. *LchiWRKY14* was highly expressed at all time points, It seems that duplication events such as tandem repeat events in the gene structure and promoter region of *WRKY* family members caused *WRKYs* to acquire diverse functions [36, 37]. The expression of *LchiWRKY27, 18, 5, 33, 23, 36* peaked at 12 h and 1d, and *LchiWRKY16, 44, 7, 37, 25, 19* were low expressed at almost all time points. We speculate that 12 h and 1d may be the main response time points, which is different from low and high-temperature stress and may be caused by different biological pathways. *LchiWRKY14, 27, 18, 5, 33, 23, 36* may be the main response genes to drought stress.

We found that some genes responded to low temperature, high temperature, and drought stress at the same time, for example, *LchiWRKY5*, *LchiWRKY23*, *LchiWRKY14*, *LchiWRKY27*, *LchiWRKY36* were up-regulated at the 1d time points in three cases, suggesting that these five genes are the main responders to abiotic stress and play across regulatory role in abiotic stress resistance, and 1d may be important time points for *LchiWRKY* to respond to abiotic stress.

### The qRT-PCR validation of *LchiWRKY* under multiple abiotic stresses

To verify the accuracy of abiotic stress transcriptome data, we performed qRT-PCR experiments of 10 genes with different expression levels, including high or low expression, *LchiWRKY7* and *44* were low expression (0 < Log2FC < 3) in the three stresses and other genes (*LchiWRKY5*, *18*, *19*, *25*, *27*, *33*, and *36*) were high expression (Log2FC > 3) at different time points of the three stresses [38] (Figs. 8, 9 and 10).

**Fig. 11:**
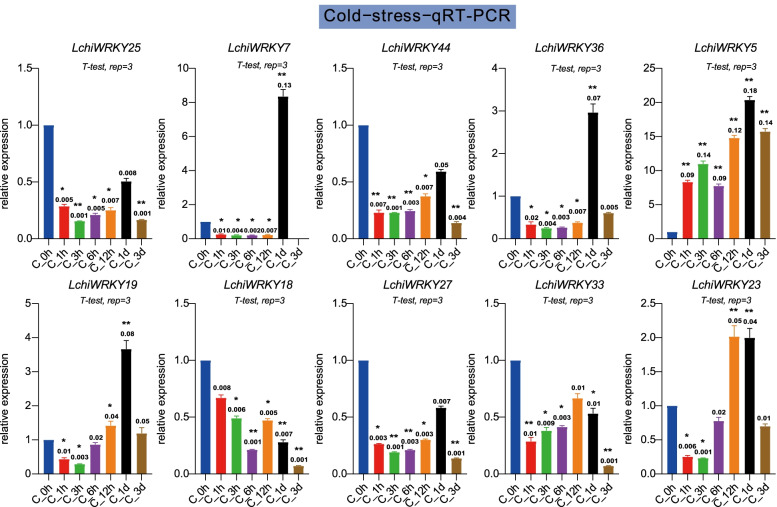
qRT-PCR verification of the cold stress response of 10 *LchiWRKYs*. Expression analysis of *LchiWRKYs* under cold stress conditions, determined by qRT-PCR. The Y-axis indicates the relative expression level and the X-axis represents different time points after stress treatment taken for expression analysis; the mean ± standard error measurement (SEM) value with three replications (*n* = 3) is displayed. Each time point was compared to the control group, T-tests were used to calculate significance: * indicates *p* < 0.05, and ** indicates *p* < 0.01

Under cold stress (Fig. 11 and Additional file 16: Table S14), *LchiWRKY7*, *36,* *19* were up-regulated and reached the peak at 1d, while *LchiWRKY23* was up-regulated and reached the peak at the 12 h time point, only *LchiWRKY5* were up-regulated at all time points. And other genes’ patterns like *LchiWRKY25, 44, 18, 27, 33* were down-regulated at all time points. As a result, these genes’ expression patterns as the same as transcriptome data, and 1d was the main response time point.

**Fig. 12:**
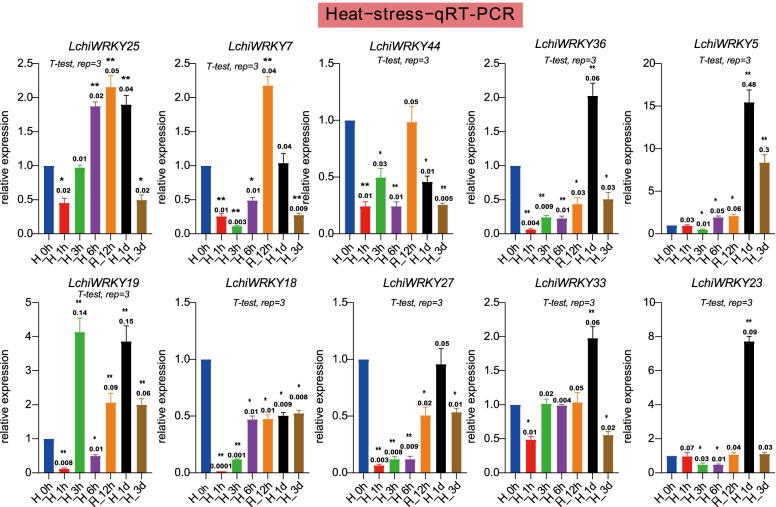
qRT-PCR verification of the heat stress response of 10 *LchiWRKYs*. Expression analysis of *LchiWRKYs* under heat stress conditions, determined by qRT-PCR. The Y-axis indicates the relative expression level and the X-axis represents different time points after stress treatment taken for expression analysis; the mean ± standard error measurement (SEM) value with three replications (*n* = 3) is displayed. Each time point was compared to the control group, T-tests were used to calculate significance: * indicates *p* < 0.05, and ** indicates *p* < 0.01

Under heat stress (Fig. 12 and Additional file 17: Table S15), the response time was earlier than that of low-temperature stress. *LchiWRKY19* was up-regulated at 3 h and reached the peak, then *LchiWRKY7, 25* were up-regulated and reached the peak at 12 h. The peak expression of *LchiWRKY36, 5, 33, 23* were at 1 d, while *LchiWRKY44, 27, 18* were low expressed at all time points. The above results showed that the expression pattern of high-temperature stress was different from that of low-temperature stress, but there was a common important response time point 1d, which indicated that this time point was the key time point for Liriodendron to respond to temperature stress.

**Fig. 13:**
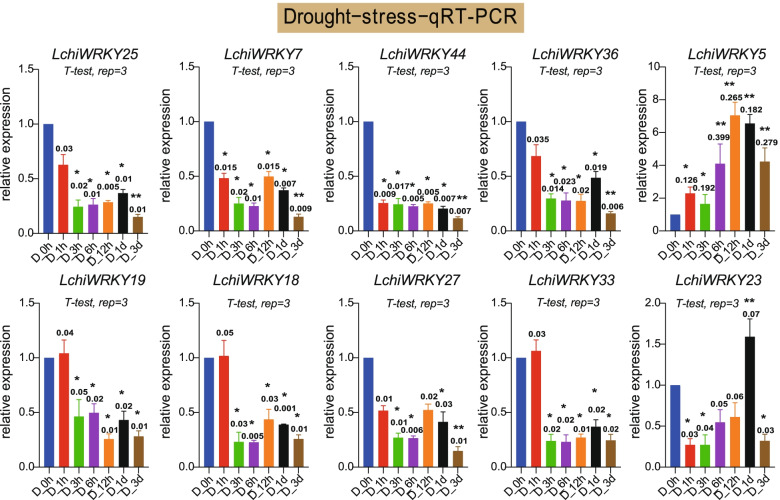
qRT-PCR verification of the drought stress response of 10 *LchiWRKYs*. Expression analysis of *LchiWRKYs* under drought stress conditions, determined by qRT-PCR. The Y-axis indicates the relative expression level and the X-axis represents different time points after stress treatment taken for expression analysis; the mean ± standard error measurement (SEM) value with three replications (*n* = 3) is displayed. Each time point was compared to the control group, T-tests were used to calculate significance: * indicates *p* < 0.05, and ** indicates *p* < 0.01

Under drought stress (Fig. 13 and Additional file 18: Table S16), the expression of most genes was relatively low than 0 h, except for *LchiWRKY5*,*23,* which had a significant peak value and were highly expressed at 12 h and 1 d, respectively. The response of *LchiWRKY18*, *19*, *33* to drought stress was earlier, peaked at 1 h, and then began to decline. *LchiWRKY25*, *7*, *44*, *36*, *27* were all low expressed after 0 h, which may be due to the inhibition of drought stress. Compared with temperature stress, the expression levels of some genes were relatively low under drought stress. For instance, *LchiWRKY7, 36* was highly expressed under both low and high-temperature stress, but low expressed under drought stress. Several genes had peak expression under three stresses, such as the expression of *LchiWRKY5, 23* peaked at 12 h and 1d of three stresses, respectively.

The expression pattern of transcriptome and qRT-PCR experiment of *LchiWRKY* gene under abiotic stress fully showed that the *WRKY* family of *L. chinense* responded to abiotic stress, which provided the theoretical basis for the adaptability promotion of *L. chinense* in different regions.

### *LchiWRKY18* and *36* were expressed in the nucleus

To understand the biological function of WRKY proteins, we performed subcellular localization analysis (Additional file 1: Table S1). The results showed that 44 *LchiWRKY* genes were expressed in the nucleus [4, 39, 40], while only *LchiWRKY42* was also expressed in the cell membrane [39, 41]. These results suggest that most LchiWRKY transcription factors were expressed in the nucleus, but may also function in other organelles, such as cell membrane. Therefore, we speculate that transcription factors mainly perform biological functions in the nucleus and regulate the expression of target genes in combination with the promoter sequence of target genes. There may also be some other situations, for example, some transcription factors (*LchiWRKY42*) may also have biological functions in the cell membrane.

**Fig. 14:**
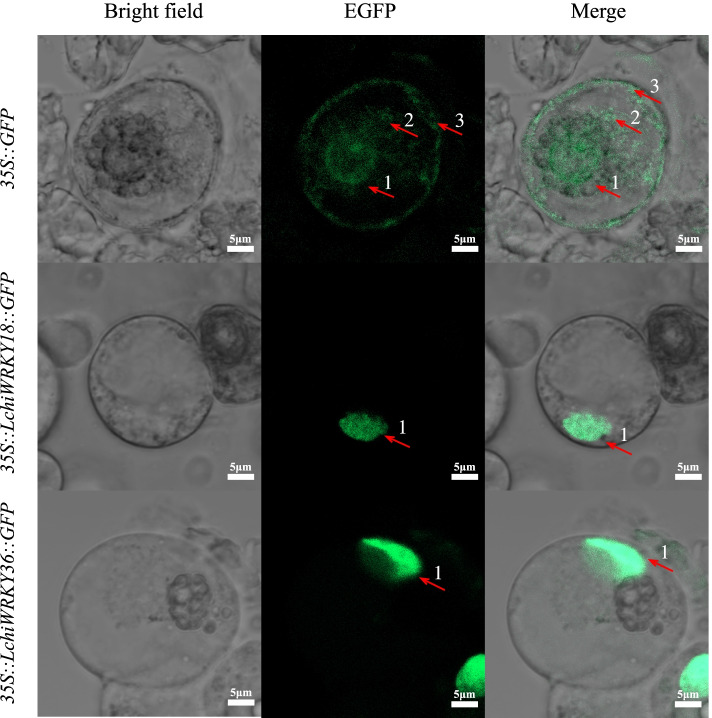
Subcellular experimental results of *LchiWRKY18* and *36*. Callus protoplasts of *Liriodendron hybrids* are used to capture bright field, green fluorescence, bright field, and green fluorescence fusion images. Red arrows 1, 2, and 3 represent the nucleus, cytoplasm, and cell membrane, respectively. Bright field indicates bright field photography, EGFP indicates green fluorescence photography, and Merge indicates the fusion of bright field and green fluorescence. Scale bar = 5 μm. *35S:: GFP* indicates the empty vector pJIT166-*GFP*, *35S::LchiWRKY18::GFP* represents recombinant vector pJIT166-*LchiWRKY18-GFP,* and *35S::LchiWRKY36::GFP* represents recombinant vector pJIT166-*LchiWRKY36-GFP*

To verify the results of subcellular localization analysis, we selected two genes *LchiWRKY18* and *LchiWRKY36* for the subcellular localization experiment. The fusion vectors pJIT166-*LchiWRKY18*-*GFP* and pJIT166-*LchiWRKY36*-*GFP* were constructed and transformed into callus protoplasts of *Liriodendron hybrids* by PEG-6000 mediated. The subcellular localization expression vector pJIT166-*GFP* was constructed as a positive control. The fluorescence of *LchiWRKY18*-*GFP* (*35S::LchiWRKY18::GFP*) fusion protein was preferentially detected in the nucleus, while the spontaneous fluorescence of the positive control pJIT166-*GFP* (*35S::GFP*) fusion protein could be detected in the whole-cell space (Fig. 14). Similarly, the fluorescence of *LchiWRKY36-GFP* (*35S::LchiWRKY18::GFP*) fusion protein was also preferentially detected in the nucleus. The results of the subcellular localization experiment show that *LchiWRKY18* and *36* are expressed in the nucleus, which is consistent with the results of subcellular localization analysis, indicating that the main biological functional position of transcription factors is in the nucleus to regulate the transcription of target genes.

## Discussion

### *Lchi* has fewer *WRKY* members, diversified biological functions and physicochemical properties, and conserved WRKY domain and motif structure

*WRKY* genes comprise a large family of transcription factors that are conserved across the whole plant kingdom. Genome-wide analysis of *WRKY* gene families has been widely carried out in a large number of species whose genomes have been sequenced [9, 42]. In the current study, a search for *WRKY* genes in the *L. chinense* genome resulted in the identification of 44 members, which we designated as *LchiWRKY1* through *LchiWRKY44*, based on their chromosomal location. Compared with some higher angiosperms, the *LchiWRKY* family has a smaller number. For example, there are 125 *ZmWRKY* genes in the maize (*Zea mays*) genome [43], 108 *HbWRKY* genes in the Rubber Tree (*Hevea brasiliensis*) genome [44], 102 *LuWRKY* genes in Flax (*Linum usitatissimum* L.) genome [45], 86 *HvWRKY* genes in Barley (*Hordeum vulgare* L.) genome [46], and 64 *IiWRKY* genes were identified in the whole genome of *Isatis indigotica* [47]. A total of 61 *CsWRKY* genes in the Cucumber (*Cucumis sativus* L.) genome [4], 57 *CmWRKY* genes were identified in the melon (*Cucumis Melo* L.) genome [48], and 54 *AcWRKYs* in Pineapple (*Ananas comosus* (L.) Merr.) genome [49]. Although the number of *WRKY* members of these plants with different evolutionary statuses is different, they are more than *L. chinense*. The results showed that the number of *WRKY* family members of *L. chinense* might be related to its evolutionary status and family expansion model, *L. chinense* was close to the basal angiosperm [23], so speculate the number of *LchiWRKY* family members might be less than that of higher angiosperm plants.

Gene ontology (GO) showed that most *WRKY* genes were enriched in molecular function, biological process, and cellular component. For instance, GO analysis of Tartary buckwheat *WRKYs* showed that most *FtWRKYs* are enriched in molecular function and biological processes, and some genes are enriched in a cellular component [41]. In Orchardgrass, 93 *DgWRKYs* were enriched in biological regulation and cellular process of biological process category, and binding and nucleic acid binding TF activity of molecular function category, while some of them were in membrane and membrane part of cellular component category [50]. In Rice, novel candidate *OsWRKY* genes were significantly enriched in sequence-specific, DNA, heterocyclic and regulatory region binding activities of molecular function [51]. In *Taraxacum antungense* Kitag, 44 *TaWRKYs* were significantly enriched in 23 terms of biological process, and cellular processes were the most representative [52]. In *L. chinense*, A total of 34 *LchiWRKYs* were significantly enriched in the two GO categories of biological processes and molecular functions, including 10 subcategories, which do not contain cellular components. This is different from the above species. It is speculated that it may be the difference in the regulatory function of WRKY transcription factors among different species. Therefore, biological processes and molecular function may be the main biological functions and processes involved in *LchiWRKYs*.

The protein length (MW) and isoelectric point (pI) of LchiWRKY proteins showed a large degree of variation across the gene family, indicating that LchiWRKYs possess different protein structures and may have adapted to different microenvironments. These features suggest that *LchiWRKYs* may have biological regulatory functions in different environments/conditions, especially during abiotic stress. The WRKY protein length, molecular weight, and isoelectric point are different in different species, for example, in Pineapple, the protein length of AcWRKYs varied from 122 to 1320 amino acid (aa), and MW varied from 13.7 to 144.5 kDa, and pI ranged from 5.11 to 10.08 [49]. In Maize, the length of ZmWRKYs varied from 99 to 729 aa, and MW varied from 11.27 to 78.7 kDa, and pI ranged from 4.55 to 10.78 [43]. Therefore, the biological functions of *WRKY* genes in different species are different, which may be related to the physical and chemical properties and spatial structure of proteins.

The conserved WRKY domains of the LchiWRKY proteins were assessed in this study. Multiple sequence alignments revealed that two LchiWRKY proteins (LchiWRKY10 and LchiWRKY37) in group IIc had sequence variation in their WRKY domain. Most characterized WRKY proteins exhibit a binding preference to their cognate *cis*-acting W-box element, with the help of the WRKY domain. According to previous studies, variations in the WRKYGQK motif within the WRKY domain might influence normal interactions of *WRKY* genes with their downstream target genes, and it might therefore be worthwhile to further investigate the binding specificity and functionality of these two WRKY proteins [53, 54]. R-type introns (PR intron) and V-type introns (VQR intron) are two major introns within the WRKY family in different plants; In *L. chinense,* the groups I, IIc, IId, III we identified contain R-type introns, and groups IIa and IIb contain V-type introns [4, 55]. These two types of introns may play important roles in biological processes, such as heat stress, cold stress, salt stress, and drought stress. For example, *LchiWRKY5 and LchiWRKY23* were highly expressed under cold, heat, and drought stress [4, 11, 55]. These WRKY protein variants and two intron insertions have different forms in different species, which indirectly reflects the conservation and diversity of *WRKY* gene in the process of plant evolution, so that the functions of *WRKY* gene among different species are not only similar but also different [4, 11, 54, 55].

The conserved motifs reveal the grouping specificity of motif distribution of *L. chinense*, a total of 20 motifs were distributed in 7 subgroups, each of which had a specific motif, except for groups IIe (LchiWRKY30) and III (LchiWRKY40, LchiWRKY42). In Orchardgrass, 10 motifs were determined and the same phylogenetic group has the same conserved motif, only group III didn’t have a C2H2 zinc finger motif [50]. In Common walnut, 15 motifs were detected in JrWRKYs, only two genes contain the CCCC domain, while other phylogenetic subgroups contain group-specific motifs [40]. The above results show that although the number of *WRKY* is different in different species, the motif has specificity in different phylogenetic groups, and the same subgroup has the same motif.

### The expansion of the *LchiWRKY* family results from tandem repeat events and functional and structural diversity of replication genes

Tandem and segmental duplication events have played a critical role in the expansion of the *WRKY * gene family [6]. In Rubber tree (*Hevea brasiliensis*), among the 25 pairs of duplicated gene pairs, 2 pairs of genes were derived from tandem duplication events and 23 pairs of genes were segmental duplication events [44]. In Quinoa (*Chenopodium quinoa* Wild.), a total of 39 pairs of replication gene pairs, 2 pairs of genes are tandem replication, 37 pairs of genes are segmental replication [14]. In Cucumber (*Cucumis sativus* L.), 14 segmental replication events occurred in 25 genes [4]. In Pineapple (*Ananas comosus*), 7 pairs of genes were tandem duplication genes and 17 segmental replication events occurred in 27 genes [49]. In Maize (*Zea mays*), 52 segmental duplication events occurred in 78 genes, and there was no tandem replication event [43]. In Barley (*Hordeum vulgare* L.), 5 tandem duplication events and 13 pairs of genes were segmental duplications [46]. In Chinese woad (*Isatis indigotica*), 5 gene pairs were tandem duplication and 7 gene pairs were segmental duplication [47]. In addition, whole-genome duplication events may also affect the number of gene families. For basal angiosperms, for example, there are no ancestor whole-genome duplication events in *A. trichopoda* [32], while there is an ancestor whole-genome duplication events in Water lily [56], which may lead to the total number of *WRKY* in water lily being more than that in *A. trichopoda* (Table 1). For Magnolia plants, two whole-genome duplication events occurred in *C. kanehirae* and one whole-genome duplication event occurred in *L. chinense* [23, 32], which may also lead to more *WRKY* in *C. kanehirae* than *L. chinense* (Table 1)*.*

A comparison of the number of *WRKY* genes in *L. chinense* with other sequenced higher angiosperm genomes shows that *L. chinense* possesses a smaller number of *WRKY* genes. This could indicate a reduced number of duplication events in *L. chinense,* only six genes had tandem replication events. Among these genes, four (*LchiWRKY14*,*15*,*20*,*27*) are from group II-d, and the other two are from group I (*LchiWRKY14*) and II-a (*LchiWRKY35*), This suggests that group IId may play a major role in the expansion of *LchiWRKY* family, and tandem repetition is the main expansion model. Therefore, the total number of gene families may be related to whole-genome duplication events, tandem duplication, and segmental duplication events, which ultimately determines the difference in the number of *WRKY* families in different species.

We found that the expression pattern of duplicated paralogous *LchiWRKY* genes was always different. Divergence of gene expression plays an important role in the preservation of duplicated genes [57, 58]. Several paralogous gene pairs show a differing response to abiotic stress, suggesting that they may play diverse roles in *L. chinense* in response to abiotic stress (Figs. 8, 9 and 10). For instance, *LchiWRKY23* was highly expressed in response to cold and drought stress at 12 h ~ 1d, while its paralogous gene *LchiWRKY35* was not induced upon cold and drought stress. Still, some *WRKY* genes and their paralogues, like *LchiWRKY14* and *LchiWRKY27*, shared similar high transcript abundance profiles under heat stress at 1d, 3d, suggesting that they may have redundant functions. Further analysis showed that the duplicated genes contained the same cis-regulatory elements (Fig. 7), such as LTR elements, which mainly responded to low-temperature stress, so they could all respond to low-temperature stress, such as *LchiWRKY27*,*14*. Combined with the results of gene structure and motif, these replicated genes also have diversity in structure and motif. For instance, LchiWRKY23 has 6 motifs and LchiWRKY35 has 7 motifs, only motif 1 is their common motif (Fig. 5). LchiWRKY27 lacks motif 18, and LchiWRKY14, 15 and 20 have it (Fig. 5). * LchiWRKY35* has 3 exons and *LchiWRKY23* has 2 exons and 4 UTRs, but no UTR is found in *LchiWRKY35* (Fig. 6).

### *LchiWRKY* gene has a cross-regulation function in response to multiple abiotic stress

According to previous studies, WRKY transcription factors are involved in abiotic stress regulation, including low temperature, high temperature, salt, and drought [4, 16, 44]. For instance, in *Ipomoea trifida, 11 Itfwrkys (Itfwrky8*, *15.1*, *22.1*, *34*, *41*, *48*, *66*, *69*, *77*, *79,* and *80*) were highly expressed under salt, drought, low temperature, and high temperature [16]. In Cucumber (*Cucumis sativus* L.), 21 *CsWRKY* genes were induced to express under heat stress, and the expression trend was the same from 3 h to 6 h, five *CsWRKY* genes (*CsWRKY27* / *41* / *50* / *52* / *57*) were induced to express under salt and heat stress [4]. In Camelina (*Camelina sativa* (L.) Crantz), the expression of *CsWRKY21* under cold stress was higher than that under drought stress, while the expression level of *CsWRKY22* under drought stress was higher than that under cold stress [17]. In Quinoa (*Chenopodium quinoa* Wild.), nearly 46 *CqWRKY* genes are induced to express under salt or drought stress. The expression level of *CqWRKY52a-1 / 2* was similar to that of group II-d, which was highly expressed under salt stress [14]. In Chickpea (*Cicer arietinum* L.), 15 genes were down-regulated in roots and 10 genes were down-regulated in stems under abiotic stress [59]. The *OsWRKY55* negatively regulates drought tolerance in transgenic rice lines [60], The transcription level of *HvWRKY42* increased in Barley (*Hordeum vulgare* L.) under drought stress, which indicated that *HvWRKY42* was regulating the drought adaptability of barley [61]. The *HvWRKY3* showed 13 fold high expression and 4 genes showed 2-fold high expression under drought stress [46]. In Maize, under drought stress, 10 *ZmWRKYs* were up-regulated in roots and 4 *ZmWRKYs* showed 10-folds expression in leaves at 8 h [43]. In Pineapple, *AcWRKY35* was induced by drought stress, and 7 *AcWRKYs* were expressed by cold stress [49]. In *Camellia japonica,* 13 *CjWRKYs* were highly expressed under salicylic acid (SA) stress for 12 h, indicating that the *CjWRKY* gene can regulate *C. japonica* to adapt to SA environmental conditions [62].

In the study, we explored the regulatory function of *LchiWRKY* under abiotic stress by treating somatic embryogenesis of *Liriodendron hybrids* seedlings with low, high-temperature, and drought stress, and the expression patterns of low temperature, high-temperature, and drought stress in the *WRKY* family were obtained. Low temperature is one of the major environmental stresses that affect *L. chinense* growth and development, and high temperature and soil drought cause the death of trees and reduce the yield of forests. According to the cold stress expression pattern (Fig. 8), the expression of 11 *LchiWRKY* genes was significantly induced. The overall expression pattern is divided into two clusters, including high expression at different time points and persistent low expression at all time points. Group I and II responded to cold stress, but group III did not respond, indicating that the response of group III to regulating cold stress was not obvious, while the regulation of groups I and II was more obvious, which also reflected the functional specificity of the group. *LchiWRKY5* and *14* may be the main members of the *WRKY* family in response to low-temperature stress, and 1d and 3d are the main response time points of the *WRKY* family. It shows that when *L. chinense* is stimulated by cold stress, it first adapts to the environment through self-protection, and then regulates its adaptability through cold stress-related resistance genes expressed at 12 h or 1d. Group II is the main cold stress regulatory gene, which promotes *L. chinense* to adapt to low-temperature environment stress.

The expression patterns of high-temperature stress were also divided into two groups (Fig. 9), including 1d and 3d high expression patterns and persistent low expression patterns. Similar to cold stress, group III was not significantly induced, and there were more response genes in group II than in group I. *LchiWRKY14*,*18* are the key genes of the *WRKY* family to respond to high-temperature stress. 1d and 3d are also the main response time points of high-temperature stress. This also shows that the adaptation of *L. chinense* to a high-temperature environment is similar to that to a low temperature. First, it carries out self-protection and then regulates its adaptability through high-temperature response genes expressed at 12 h or 1d. Group II is also the main regulatory gene of *L. chinense* in response to high-temperature stress, which indicates that the function of group II is very important for temperature stress.

The expression patterns of drought stress can also be divided into two groups, containing a high expression pattern of 12 h, 1d, and a persistent low expression pattern (Fig. 10). Different from low temperature and high-temperature stress, group III genes were significantly induced, indicating that group III may be drought-specific response genes. The number of genes in groups I and II are still more than that in group III, indicating that these two groups are also important genes in response to drought stress. *LchiWRKY14*,*18* may be the main response gene and 12 h, 1d may be the main response time point to drought stress. This shows that *L. chinense* adapts to drought stress as well as temperature stress. It first protects itself and then adapts to the environment by specifically regulating gene expression at 12 h or 1d. *LchiWRKY* family can regulate *L. chinense* to adapt to drought environment, but the regulation function of groups I and II are more obvious than that of group III.

Interestingly, some *WRKY* genes have high expression patterns under different stresses, such as *LchiWRKY5*, *23*, *14*, *27*, *36*, which are highly expressed at 1d time points of three stresses, indicating that these genes may have a cross-regulation function under different stresses. These five *LchiWRKYs* were also the key regulatory genes for the growth and development of *L. chinense* to promote *L. chinense* to adapt to different adverse environmental conditions. The results of qRT-PCR under low, high-temperature, and drought stress also showed that 1d was the main response time point of the *WRKY* family, which was consistent with the results of the transcriptome, and reflected the accuracy of transcriptional data.

### *LchiWRKY* transcription factor is mainly expressed in the nucleus

As transcription factors, they are mainly expressed in the nucleus [40, 49–51, 63], but some are also expressed in other organelles, such as chloroplast, mitochondria, cytoplasm, and cell membrane [43, 51, 63]. Subcellular localization analysis of maize showed that most ZmWRKY transcription factors were expressed in the nucleus, and only a few *ZmWRKYs* were expressed in other organelles [43]. In pineapple, 51 *AcWRKYs* were located in the nucleus and only 3 in the chloroplast [49]. Similarly, in Orchardgrass, most of *DgWRKYs* (88.17%) were located in the nucleus, and only a few in chloroplasts (7), cytoplasm (2), mitochondria (1), and peroxisome (1) [50]. The difference is that in walnut, all *JrWRKYs* were expressed in the nucleus [40]. The Subcellular localization analysis of *L. chinense* showed that all *LchiWRKYs* were expressed in the nucleus, except the *LchiWRKY42* was also expressed in the cell membrane. Combined with phylogenetic tree (Fig. 1) analysis, *LchiWRKY42* is in a separate branch, so it is speculated that its function is different from other genes, which may be due to functional differentiation in the process of evolution.

The subcellular localization experiments of *LchiWRKY18* and *LchiWRKY36* in *L. chinense* showed that these two transcription factors were located in the nucleus (Fig. 14), indicating that they were expressed in the nucleus and regulated the expression of target genes by binding the promoter sequence of target genes. In other species, the subcellular localization experiments of WRKY transcription factor are mainly in the nucleus, For example, in Sugarcane (*Saccharum* spp.), the subcellular localization experiment of *ScWRKY5* showed that it was expressed in the nucleus [64]. In sandalwood (*Santalum album* L.), the SaWRKY1 protein was also expressed in the nucleus [39]. In *Liriodendron hybrids,* the LhWOX1 protein was expressed in the nucleus [65].

Based on the above analysis, the expression position of WRKY transcription factor is different in different species, but most members are mainly expressed in the nucleus and a few in other organelles, indicating that the main function of WRKY transcription factors is to regulate gene expression, but also participate in other biological processes.

Overall, the above findings provide insight into the potential functional role of *L. chinense WRKY* genes. The *LchiWRKY* family has a cross-regulation function, which makes *L. chinense* adapt to different stress environments. Our comprehensive analyses helped select candidate *WRKY* genes for further functional verification and genetic improvement of *L. chinense* agronomic traits and environmental resistance. Additionally, to provide a theoretical basis for promoting afforestation of Liriodendron in different areas.

## Conclusions

A comprehensive analysis of the *WRKY* gene family in *L. chinense* was carried out in the present study. Forty-four full-length *WRKY* genes were characterized and further classified into three main phylogenetic groups, phylogenetic comparison of *WRKY* genes from 17 different plant species provided valuable clues about the evolutionary characteristics of *LchiWRKY* genes, and *L. chinense* has fewer *WRKY* members than other higher angiosperms. *LchiWRKY* family has a highly similar exon-intron structure and motif compositions within the same subgroup and the regulatory functions of different subgroups are specific. Subcellular localization analysis and experiments showed that *LchiWRKYs* were mainly expressed in the nucleus, and GO analysis focused on biological processes and molecular functions. The analysis of gene replication events and selection pressure showed that the expansion of the *WRKY* family was mainly through tandem repeat events, and suffered from purification selection during evolution, which may lead to a small number of *WRKY* members. More importantly, *LchiWRKY* is also involved in the regulation of abiotic stress, *LchiWRKY5*, *23*, *14*, *27,* and *36* cross-regulate the response to low, high-temperature, and drought stress. These results provide a valuable resource for a better understanding of the biological roles of *WRKY* genes in *L. chinense.* At the same time, it also provides a theoretical basis for the growth and development and afforestation in different areas of Liriodendron.

## Methods

### Datasets and sequence retrieval

The complete genome, transcript/protein sequences, and genome feature file of *L. chinense* (*Lchi*) were downloaded from https://www.ncbi.nlm.nih.gov/assembly/GCA_003013855.2. All 16 species contained basal angiosperms *Amborella trichopoda, Nymphaea colorata, Magnolia Cinnamomum kanehirae*, monocotyledons (*Brachypodium distachyon*, *Panicum hallii*, *Oryza sativa, Spirodela polyrhiza*), dicotyledons (*Arabidopsis thaliana*, *Capsella grandiflora*, *Cucumis sativus*, *Eucalyptus grandis*, *Malus domestica*, *Gossypium raimondii*, *Populus trichocarpa*, *Theobroma cacao*, *Vitis vinifera),* WRKY proteins were obtained from the Phytozome 13 (https://phytozome-next.jgi.doe.gov/), with search in ‘WRKY’ keyword for these plant species, respectively. Each sequence was checked manually, and the protein sequence without the WRKY domain was deleted.

An HMM (Hidden Markov Model) profile for the WRKY (PFAM ID: PF03106.15) domain (SMART ID: SM00774 and InterPro ID: IPR003657) was retrieved from Pfam (http://pfam.xfam.org) and then used as a query to search all the WRKY domain-containing protein sequences in the *L. chinense* genome using HMMER (v.3.0.1b) with an E-value cut of < 1E-5. Finally, all candidate LchiWRKYs were validated using the Pfam and the Conserved Domains Database (CDD, https://www.ncbi.nlm.nih.gov/Structure/cdd/wrpsb.cgi) to determine that they indeed contained the core domain sequences.

### Sequence analysis

The gene structure information of each *LchiWRKY* gene was acquired from the genomic feature file (GFF3) and displayed using GSDS_2.0_ (http://gsds.gao-lab.org/index.php) [66], while the chromosomal location and microsynteny of *LchiWRKYs* were visualized using the R package Circlize [67] (https://cran.r-project.org/web/packages/circlize/). The Multiple Collinearity Scan toolkit (MCScanX) program was used to verify putative paralogous genes (blast hits E-value cutoff<1E-6, collinearity > 70%) [68]. The *cis*-acting elements of *LchiWRKY* genes were analyzed by PlantCARE (http://bioinformatics.psb.ugent.be/webtools/plantcare/html/) and displayed using TBtool software [69]. Ka/Ks values were calculated using the Ka/Ks_calculator [70]. Basic properties of LchiWRKY proteins, including length, molecular weight (MW), and isoelectric point (pI) were calculated using ExPasy (http://au.expasy.org/tools/pi_tool.html) and then subcellular localization was analyzed by Plant-mPLoc website (http://www.csbio.sjtu.edu.cn/bioinf/plant-multi/) [71, 72]. The conserved motifs of LchiWRKY proteins were predicted using MEME(v5.4.1) (https://meme-suite.org/meme/tools/meme) with the following settings: the discovery mode was classic, site distribution was zero or one occurrence (of a contributing motif site) per sequence, the background is 0-order background model, the maximum number of different motifs: 20, minimum motif width: 6, and maximum motif width: 50 [35], and displayed using the TBtool software [69]. Gene ontology (GO) analysis was implemented by the clusterProfiler(v4.0.5) R package with *LchiWRKY* GO id annotated against all GO id of all *Lchi* genes (Additional file 22: Table S19), in which gene length bias was corrected. GO terms with corrected *P*-value less than 0.05 were considered significantly enriched by *LchiWRKY* genes [73].

### Phylogenetic analysis

Multiple sequence alignment (MSA) of *Amborella trichopoda*, *Liriodendron chinense*, *Brachypodium distachyon*, *Cinnamomum kanehirae, Gossypium raimondii*, *Oryza sativa*, *Arabidopsis thaliana*, *Capsella grandiflora*, *Cucumis sativus*, *Eucalyptus grandis*, *Malus domestica*, *Nymphaea colorata, Spirodela polyrhiza, Panicum hallii*, *Populus trichocarpa*, *Theobroma cacao*, *Vitis vinifera* WRKY domain-containing full-length proteins with FASTA format was done using MUSCLE (v3.8.31) set the following parameters: the maximum number of iterations was 1 with ‘-maxiters 1’, and find diagonals with ‘-diags -sv -distance1 kbit20_3’ [74]. The trimmed MSA file was generated with trimAl (v1.4) [75] set to ‘automated1’ mode and then used to construct the WRKY phylogenetic tree. The Bayesian phylogenetic tree was constructed using the BEAST software (v2.6.6), by inputting the trimmed file in FASTA format with BEAUti 2 program under the following settings: the Site model was Gamma Site model, substitution rate was 1.0, and substitution model was Dayhoff of site model, clock.rate of clock model was 1.0, Priors of the tree was set to the Yule Model, the Birth Rate was 1.0, the MCMC chain length was 10,000,000, and then output the file in XML format. After the BEAST program is completed and the TreeAnnotator program set posterior probability limit was 1.0 with a Burnin percentage of 90, target tree type was maximum clade credibility tree, node heights were common ancestor heights to build the Bayesian phylogenetic tree (the Bayesian tree file was shown in Additional files 2 and 3: Tables S2-S3) [76]. The phylogenic tree was then visualized using the R package ggtree [77, 78] (http://www.bioconductor.org/packages/release/bioc/html/ggtree.html). OrthoFinder (v2.2.3) was used to explore the WRKY evolutionary status of all the 17 species by using a folder of their protein full-length sequences in the FASTA format with the parameter ‘-f’, and used the DIAMOND sequence search and MSA gene tree inference, then MAFFT was used for multiple sequence alignment and the IQ-TREE program was used to construct species tree with maximum likelihood species tree inference, and the 17 rooted species tree file was shown in (Additional file 4: Table S4) [79–81], and then displayed the rooted species tree by using the R package ggtree [77].

### Plant materials and stress treatment

*Liriodendron hybrids* seedlings generated through somatic embryogenesis were used as the starting material throughout this study. Before any experiments were performed, plantlets were taken out of the culture medium vessel and acclimatized in a greenhouse for 2 weeks (22 °C, long day photoperiod of 16 h light/8 h dark and 75% relative humidity). For various abiotic stress treatments, plants were transferred to a growth chamber (long time photoperiod of 16 h light/8 h dark and 75% relative humidity): to simulate cold or heat or drought stress, plantlets were subjected to a 4 °C or 40 °C or 15% PEG6000 (the 15% concentration was determined according to the reports of references [47, 49, 82, 83] and the preliminary experimental results (Additional file 19: Fig. S3), the control group was added the same amount of water to the substrate and the temperature was 22 °C) treatment respectively for 1 h, 3 h, 6 h, 12 h, 1d, and 3d in the growth chamber. Each treatment consisted of five biological replicates (five plantlets) for each time point. All treated leaf tissue samples of each plantlet were immediately frozen in liquid nitrogen and stored at − 80 °C for further RNA extraction.

### RNA extraction, library preparation, and sequencing

RNA degradation and contamination were monitored on 1% agarose gels. RNA purity was checked using the NanoPhotometer® spectrophotometer (IMPLEN, CA, USA).RNA integrity was assessed using the RNA Nano 6000 Assay Kit of the Bioanalyzer 2100 system (Agilent Technologies, CA, USA), cDNA was synthesized using a HiScript® III 1st Strand cDNA Synthesis Kit (Vazyme, Nanjing, China) with the extracted RNA as a template.

A total amount of 1 μg RNA per sample was used as input material for the RNA sample preparations. Sequencing libraries were generated using NEBNext® UltraTM RNA Library Prep Kit for Illumina® (NEB, USA) following the manufacturer’s recommendations and index codes were added to attribute sequences to each sample. Briefly, mRNA was purified from total RNA using poly-T oligo-attached magnetic beads. Fragmentation was carried out using divalent cations under elevated temperature in NEBNext First Strand Synthesis Reaction Buffer (5X). First-strand cDNA was synthesized using random hexamer primer and M-MuLV Reverse Transcriptase (RNase H-). Second strand cDNA synthesis was subsequently performed using DNA Polymerase I and RNase H. Remaining overhangs were converted into blunt ends via exonuclease/polymerase activities. After adenylation of 3’ends of DNA fragments, NEBNext Adaptor with hairpin loop structure was ligated to prepare for hybridization. To select cDNA fragments of preferentially 250 ~ 300 bp in length, the library fragments were purified with AM Pure XP system (Beckman Coulter, Beverly, USA). Then 3 μl USER Enzyme (NEB, USA) was used with size-selected, adaptor-ligated cDNA at 37 °C for 15 min followed by 5 min at 95 °C before PCR. Then PCR was performed with Phusion High -Fidelity DNA polymerase, Universal PCR primers, and Index (X) Primer. At last, PCR products were purified (AMPure XP system) and library quality was assessed on the Agilent Bioanalyzer 2100 system.

The clustering of the index-coded samples was performed on a cBot Cluster Generation System using TruSeq PE Cluster Kit v3-cBot-HS (Illumia) according to the manufacturer’s instructions. After cluster generation, the library preparations were sequenced on an Illumina Novaseq 6000 platform and 150 bp paired-end reads were generated.

### Transcriptome (RNA-seq) analysis

The *L. chinense* 7 time points leaf transcriptome with biological triplicates (three plantlets) of heat, cold, and drought stress was analyzed using RNA-seq pipeline, Raw data (raw reads) of FastQ format files were firstly carried out quality control through FastQC (v0.11.9) to obtain the overview of Raw data, including Basic Statistics, Per base sequence quality, Per sequence quality scores, Per sequence GC content, Per base N content, Sequence Length Distribution, and Adapter Content. Then the Raw data were filtered according to the quality control results by using Trimmomatic (v0.39) with the following parameters: remove adapters (ILLUMINACLIP: TruSeq3-PE.fa:2:30:10), remove leading low quality or N bases (below quality 10) (LEADING:10), remove trailing low quality or N bases (below quality 10) (TRAILING:10), scan the read with a 4-base wide sliding window, cutting when the average quality per base drops below 20 (SLIDINGWINDOW:4:20), drop reads below the 90 bases long (MINLEN:90) [84]. In this step, clean data (clean reads) were obtained by removing reads containing adapter, reads containing ploy-N and low-quality reads from Raw data. At the same time, Q20, Q30, and GC content of the clean data were calculated. All the downstream analyses were based on clean data with high quality.

Reference genome and gene model annotation files *L. chinense,* were downloaded from the NCBI website (https://www.ncbi.nlm.nih.gov/assembly/GCA_003013855.2), index of the reference genome was built using Hisat2 (v2.2.1) and paired-end clean reads were aligned to the reference genome using Hisat2 (v2.2.1) [85]. We selected Hisat2 as the mapping tool for that Hisat2 can generate a database of splice junctions based on the gene model annotation file and thus a better mapping result than other non-splice mapping tools. Then we used the Kallisto (v0.46.1) to quantify gene expression by calculating all clean reads mapped to this gene with “kallisto quant” command, and count Transcripts Per Million (TPM) values of individual genes, considering the effect of sequencing depth and gene length for the reads count at the same time and is currently the most commonly used method for estimating gene expression levels [86]. We used DESeq2 (v1.16.1) with the (ANOVA method: p.adjust< 0.05, |Log2FC| > 1) to determine differentially expressed genes (DEGs), and TPM values were used to calculate the transcript abundance of *LchiWRKYs*. All *L. chinense* genes had been annotated previously in the Pfam database (http://pfam.xfam.org/) and Blast2GO website (http://www.blast2go.com). The data quality, mapping rate, gene annotation, and normalization (TPM values) of heat, cold, and drought stress RNA-seq are shown in (Additional files 20, 21, 22, and 23: Tables S17–S20). Expression heatmaps were created using the pheatmap (v1.0.12) R package, based on the transformed data of log2 (TPM + 1) values. The transcriptome data used in this study has been archived and can also be obtained on the NCBI website, cold and heat stress accession numbers were PRJNA679089 (https://www.ncbi.nlm.nih.gov/bioproject/PRJNA679089/), and drought stress accession number was PRJNA679101 (https://www.ncbi.nlm.nih.gov/bioproject/PRJNA679101/).

### qRT-PCR analysis

Primers used for quantifying the expression of *LchiWRKYs* were designed by using the primer3 website (https://www.yeastgenome.org/primer3) (Additional file 24: Table S21). qRT-PCR was carried out with a Roche Lightcyler® 480II instrument using 2x AceQ® qPCR SYBR® Green Master Mix (Without ROX) (Vazyme, Nanjing, China). The composition of the PCR mix was as follows: 10 μl 2x AceQ® qPCR SYBR® Green Master Mix (Without ROX), 0.4 μl of each primer and 1 μl of cDNA template (10 ng/μl), adding 8.2 μl ddH_2_O in a final volume of 20 μl. The housekeeping *L. chinense 18S* gene was used as an internal control. The melt and standard curve of *WRKY* qRT-PCR primers were shown in Additional file 25: Fig. S4. The reaction was carried out as follows: 95 °C for 10 min, followed by 45 cycles of 95 °C for 10 s, then 60 °C for 30 s. All reactions were run in 96-well plates. Each reaction was performed in biological triplicates, as well as three technical replicates. All data generated from real-time PCR amplification was analyzed using a 2 − ^△△CT^ method.

### Subcellular localization experiment of *LchiWRKY18* and *LchiWRKY36*

The RNA extracted from the leaves of *Liriodendron hybrids* was reversed into cDNA, and the full-length sequences of *LchiWRKY18* and *36* CDS were cloned. The *LchiWRKY18* and *36* sequences were recombined by digesting the HindIII and XbaI sites of empty vector pJIT166-*GFP* to obtain pJIT166-*LchiWRKY18*-*GFP* and pJIT166-*LchiWRKY36*-*GFP* fusion expression vectors. The primers of the *LchiWRKY18* recombination vector were 5′-atttggagaggacagcccaagcttCATGCTTAGAATGGAGGACTCAC-3′(forward direction) and 5′-ccttgctcaccatggatcctctagaCAAGAGGGAAGAACAAGGAT-3′(the reverse direction). The primers of the *LchiWRKY36* recombination vector were 5′-atttggagaggacagcccaagcttCATGGAGCAGTTGATCTTTATGTT-3′(forward direction) and 5′-ccttgctcaccatggatcctctagaCAAGGGTCGAACACGAG-3′(the reverse direction). The vector map of pJIT166-*GFP,*pJIT166-*LchiWRKY18*-*GFP,* and pJIT166-*LchiWRKY36*-*GFP* were in Additional file 26: Fig. S5.

The callus of *Liriodendron hybrids* cultured for 20 days was used to prepare protoplasts, and protoplasts were slowly and gently dissolved into a solution (10 mL) containing 0.5Mmannitol, 20 mM MES, pH 5.7, 20 mM KCl, 0.1% (w/v) bovine serum albumin, 10 mM CaCl_2._ and digested at 28 °C under dark conditions for 3 h [65], The protoplasts were transformed by PEG-6000, pipetted into a 6-well cell culture plate, and cultured at 23 °C under dark for 16 ~ 48 h [65], then the fluorescence effect of protoplasts was observed by ZEISS LSM 800 fluorescence microscope (Carl Zeiss, Germany).

## Supplementary Information

**Additional file 1: Table S1.** Physicochemical properties and chromosomal details of LchiWRKY proteins.

**Additional file 2: Table S2. ***LchiWRKYs* Bayesian Phylogenetic tree.

**Additional file 3: Table S3.** 1308 WRKY protein sequences and Bayesian phylogenetic tree of the 17 species.

**Additional file 4: Table S4.** The Gene ID and corresponding subgroup information and rooted species tree of *WRKY* family of 17 species.

**Additional file 5: Table S5.** Chromosome length and location of *LchiWRKYs*.

**Additional file 6: Table S6.** List of tandem replicated *LchiWRKYs*.

**Additional file 7: Table S7.** Motifs identified in the LchiWRKYs.

**Additional file 8: Figure S1.** GO enrichment analysis of *LchiWRKY* genes.

**Additional file 9: Table S8.** Number and length of *LchiWRKY* exons and introns.

**Additional file 10: Figure S2.** Multiple sequence alignment of LchiWRKYs.

**Additional file 11: Table S9.** Domain sequence and groups of LchiWRKYs.

**Additional file 12: Table S10.** List of cis-regulatory elements (CREs) present in the promoter region of identified *LchiWRKYs*.

**Additional file 13: Table S11. ***LchiWRKY’s* TPM value of cold stress.

**Additional file 14: Table S12. ***LchiWRKY’s* TPM value of heat stress.

**Additional file 15: Table S13. ***LchiWRKY’s* TPM value of drought stress.

**Additional file 16: Table S14.** Standardization data of qRT-PCR conditions for cold stress.

**Additional file 17: Table S15.** Standardization data of qRT-PCR conditions for heat stress.

**Additional file 18: Table S16.** Standardization data of qRT-PCR conditions for drought stress.

**Additional file 19: Figure S3.** 15% PEG6000 pre-experimental phenotype.

**Additional file 20: Table S17.** RNA-seq quality data for three stress experiments.

**Additional file 21: Table S18.** Mapping rate of reference genome used for RNA-seq of three stress experiments.

**Additional file 22: Table S19.** Genome-wide gene Pfam and GO annotation of *Lchi*.

**Additional file 23: Table S20.** All DEGs TPM values from three stress experiments.

**Additional file 24: Table S21.** List of primers used in the qRT-PCR analysis for the selected *LchiWRKYs*.

**Additional file 25: Figure S4.** Melt and standard curve of qRT-PCR primers of *LchiWRKYs*.

**Additional file 26: Figure S5.** Three fusion expression vector Maps.

## Data Availability

All data analyzed during this study are included in this article and its additional files.

## Abbreviations

*Lchi*: *Liriodendron chinense*
WD: WRKY Domain
AtWRKY: *A. thaliana* WRKY
AmtrWRKY: *A. trichopoda* WRKY
BdWRKY: *B. distachyon* WRKY
CgWRKY: *C. grandiflora* WRKY
CsWRKY: *C. sativus* WRKY
CkWRKY: *C. kanehirae* WRKY
EgWRKY: *E. grandis* WRKY
GraWRKY: *G. raimondii* WRKY
LchiWRKY: *L. chinense* WRKY
MdWRKY: *M. domestica* WRKY
NcWRKY: *N. colorata* WRKY
OsWRKY: *O. sativa* WRKY
PhWRKY: *P. hallii* WRKY
PtrWRKY: *P. trichocarpa* WRKY
SpWRKY: *S. polyrhiza* WRKY
TcWRKY: *T. cacao* WRKY
VvWRKY: *V. vinifera* WRKY
TFs: Transcription factors
WGD: Whole-genome duplication
qRT-PCR: Quantitative real-time PCR
GO: Gene ontology
MW: Protein molecular weight
pI: Isoelectric point
aa: Amino acids
MSA: Multiple sequence alignment
DEGs: Differentially Expressed Genes
TPM: Transcripts Per Million
SA: Salicylic Acid
ABA: Abscisic Acid
HMM: Hidden Markov Model
FPKM: Fragments Per Kilobase of exon model per Million mapped fragments
PIN: PIN-FORMED
GAox: Gibberellin oxidase
CBF: C-regeneration binding factors
Al: Aluminum
GAD: Glutamate decarboxylase
MYB: Myeloblastosis
