# Supplementary figures and images for: Genome-wide identification of the Liriodendron chinense WRKY gene family and its diverse roles in response to multiple abiotic stress
Source: BMC Plant Biol. 2022 Jan 10;22:25. doi: 10.1186/s12870-021-03371-1 (PMC8744262; doi:10.1186/s12870-021-03371-1)

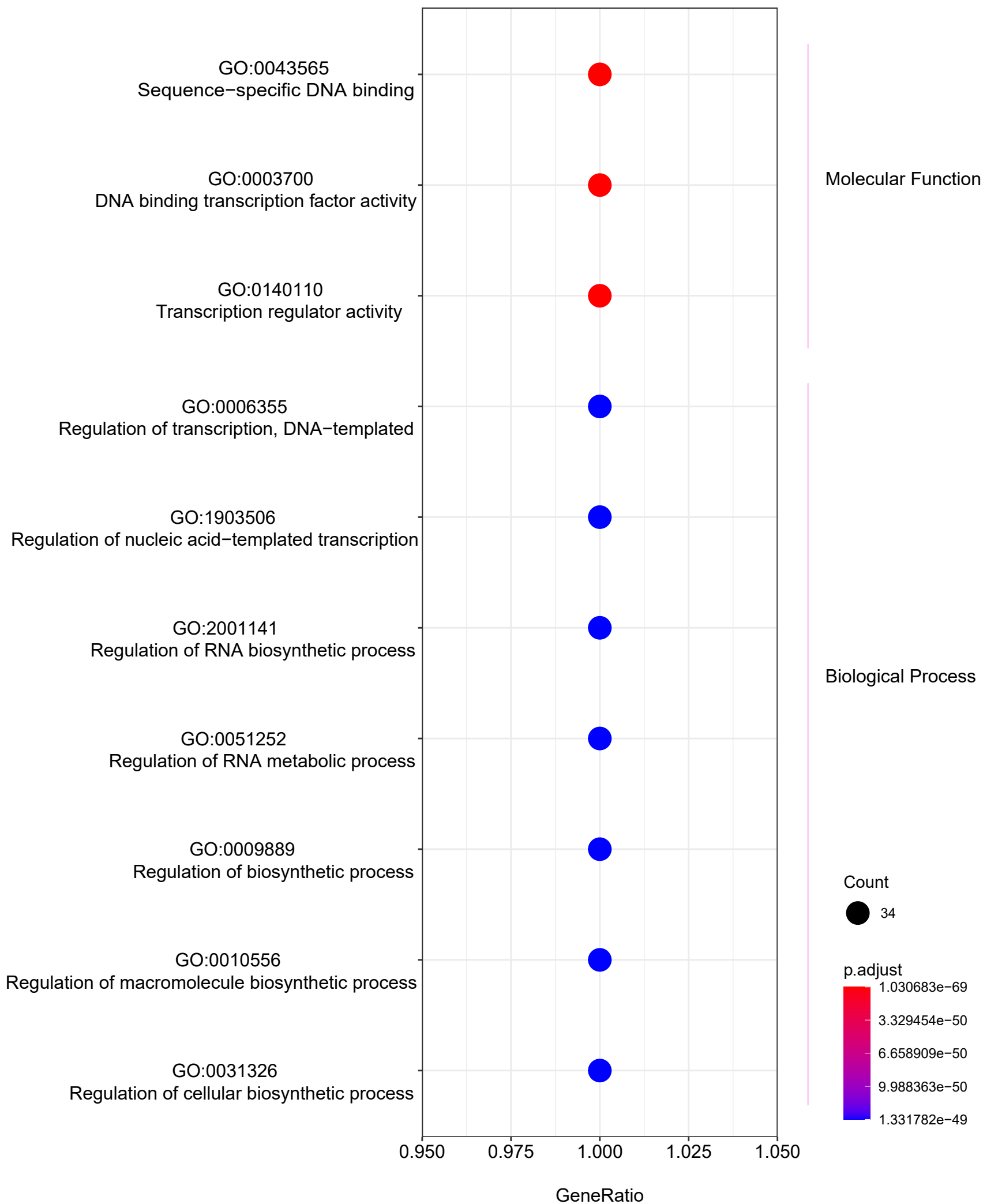

Supplement: Supplementary file 8 — Additional file 8: Figure S1. GO enrichment analysis of LchiWRKY genes. [file 12870_2021_3371_MOESM8_ESM.pdf]

Group II-a, II-b

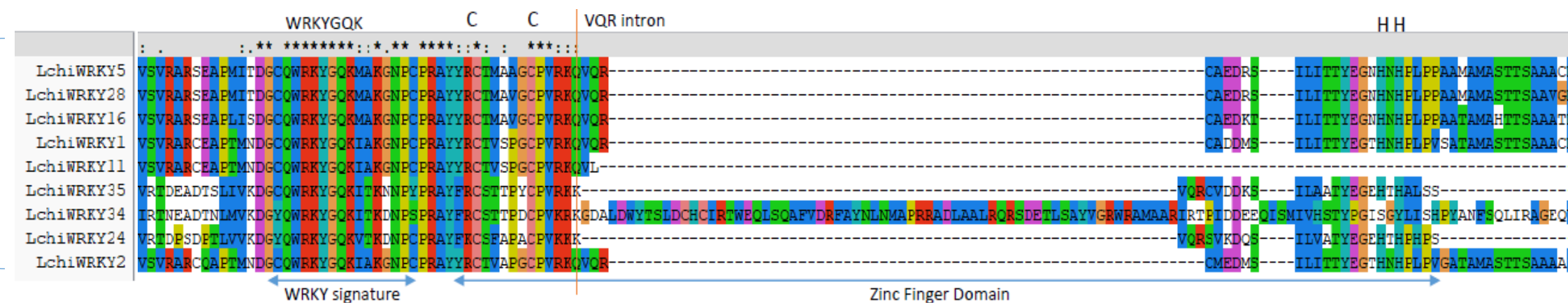Group I, II-c,  
II-d, II-e, III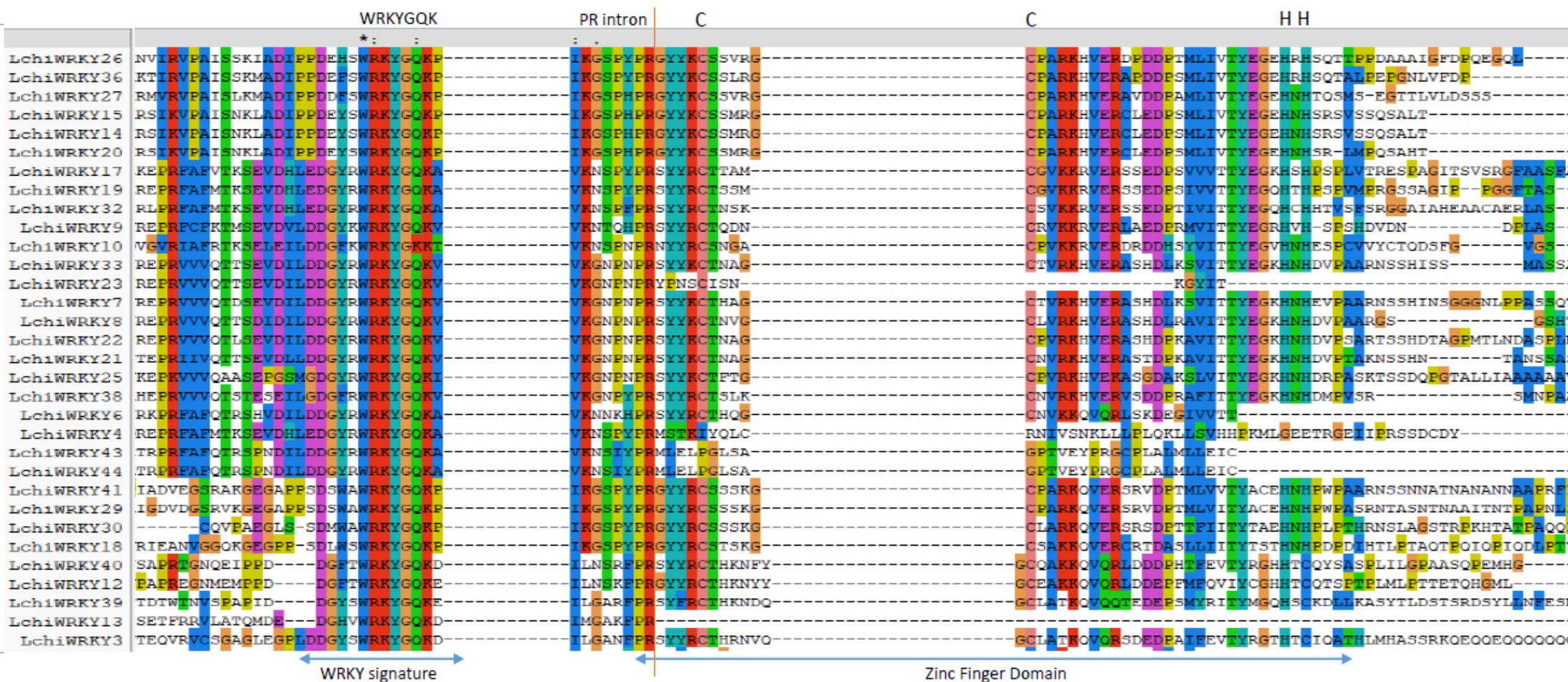

Supplement: Supplementary file 10 — Additional file 10: Figure S2. Multiple sequence alignment of LchiWRKYs. [file 12870_2021_3371_MOESM10_ESM.pdf]

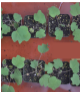

10% PEG6000 CK

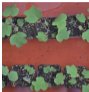

10% PEG6000 1d

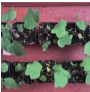

10% PEG6000 3d

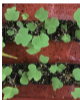

15% PEG6000 CK

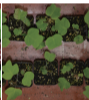

15% PEG6000 1d

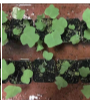

15% PEG6000 3d

Supplement: Supplementary file 19 — Additional file 19: Figure S3. 15% PEG6000 pre-experimental phenotype. [file 12870_2021_3371_MOESM19_ESM.pdf]

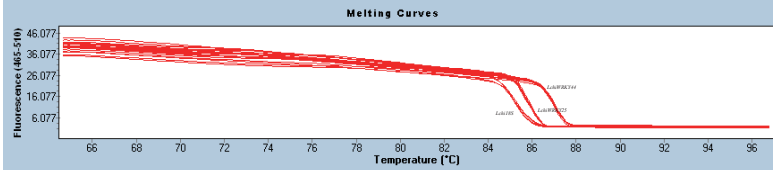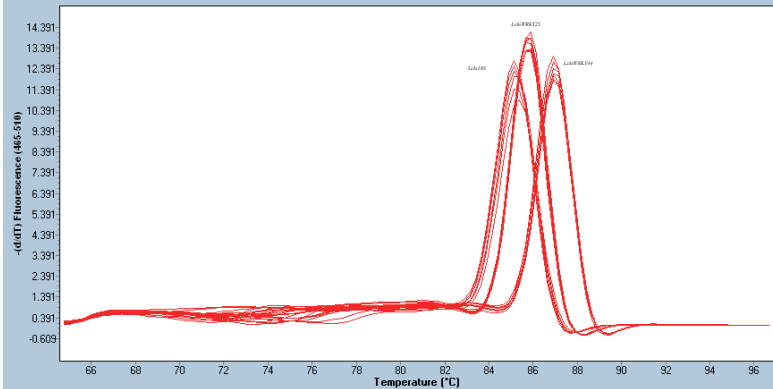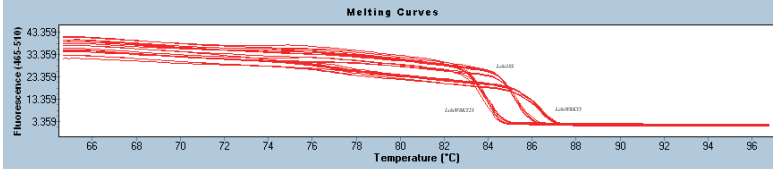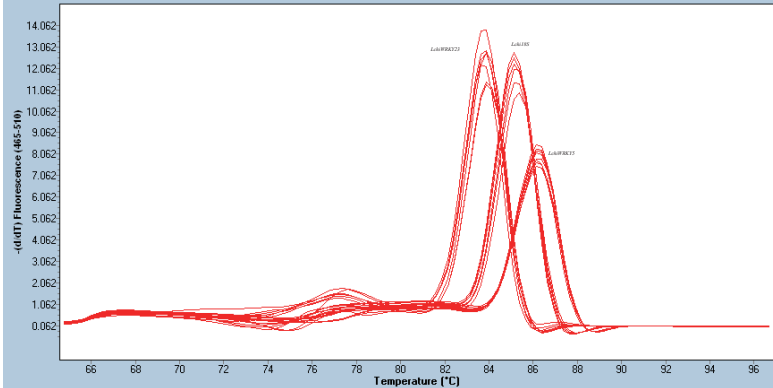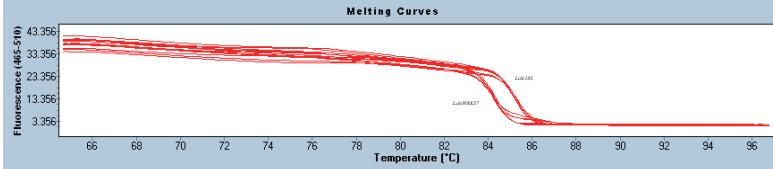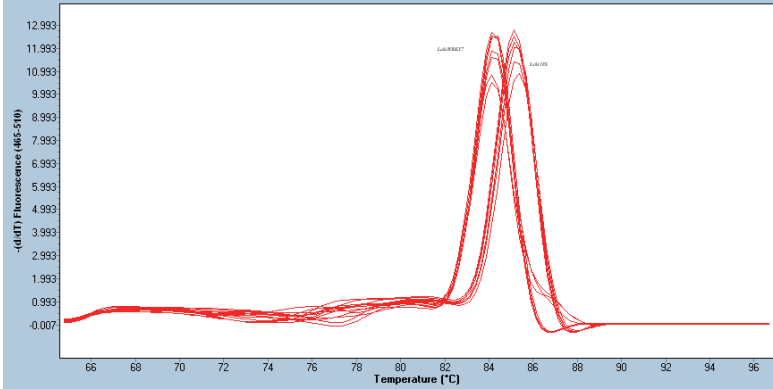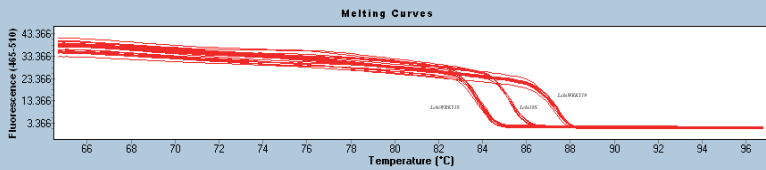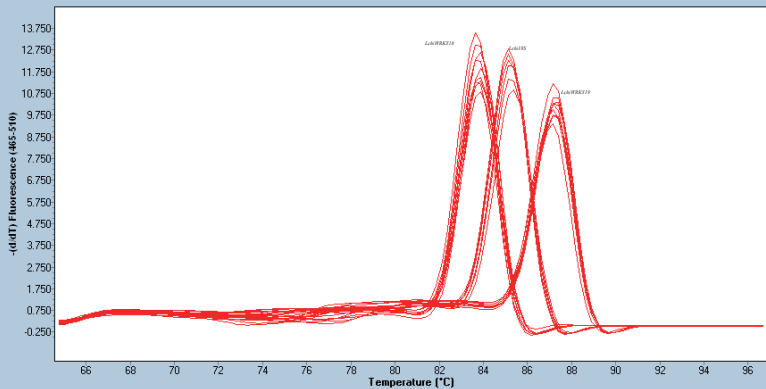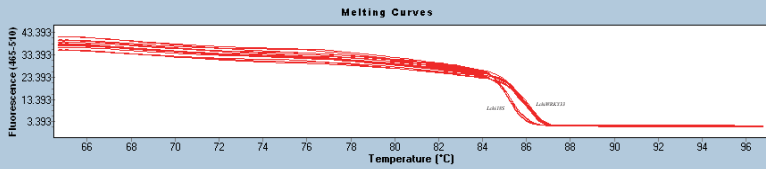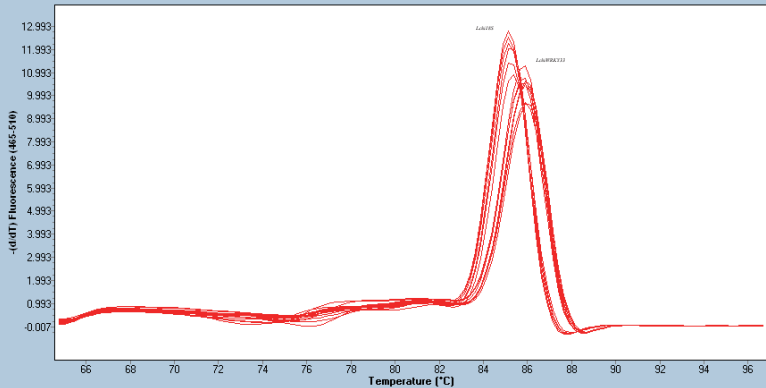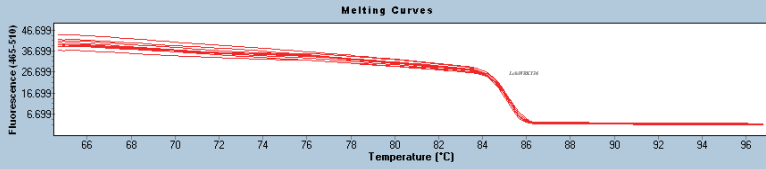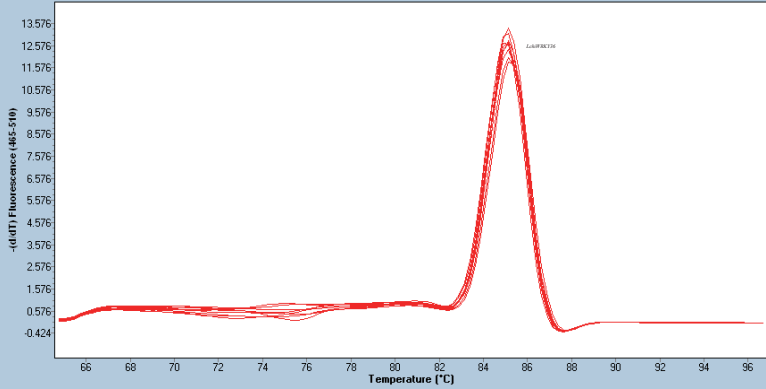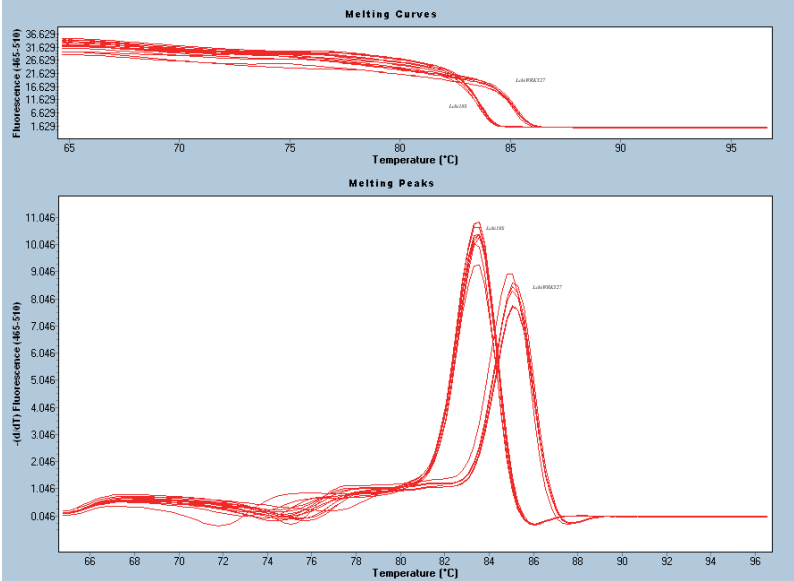

Supplement: Supplementary file 25 — Additional file 25: Figure S4. Melt and standard curve of qRT-PCR primers of LchiWRKYs. [file 12870_2021_3371_MOESM25_ESM.pdf]

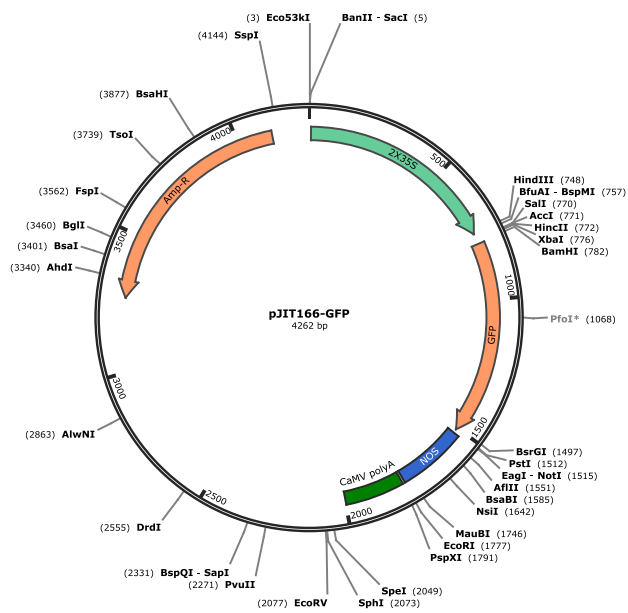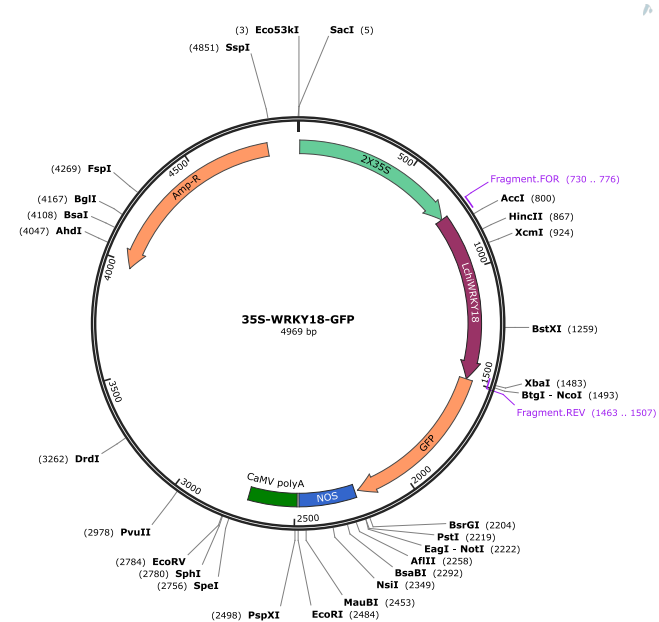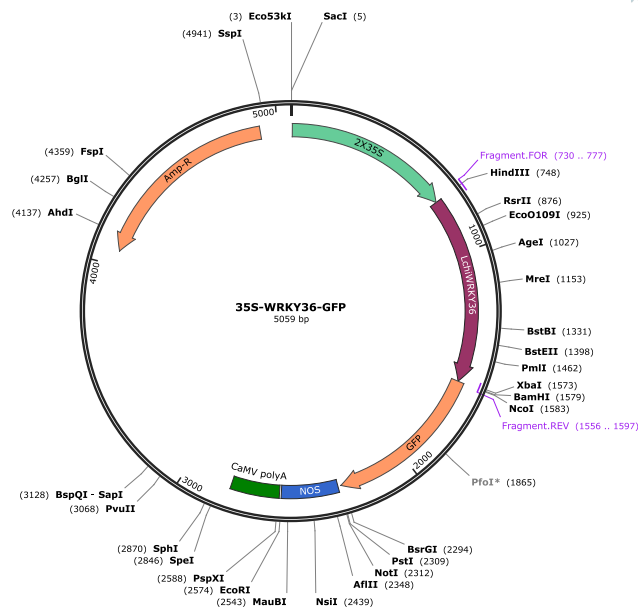

Supplement: Supplementary file 26 — Additional file 26: Figure S5. Three fusion expression vector Maps. [file 12870_2021_3371_MOESM26_ESM.pdf]
